# Vision-Based Localization in Urban Areas for Mobile Robots

**DOI:** 10.3390/s25041178

**Published:** 2025-02-14

**Authors:** Erdal Alimovski, Gokhan Erdemir, Ahmet Emin Kuzucuoglu

**Affiliations:** 1 Computer Engineering Department, Istanbul Sabahattin Zaim University, 34303 Istanbul, Türkiye; erdal.alimovski@izu.edu.tr; 2 Department of Engineering Management and Technology, University of Tennessee at Chattanooga, Chattanooga, TN 37405, USA; 3 Department of Electrical and Electronics Engineering, Faculty of Technology, Marmara University, 34722 Istanbul, Türkiye; kuzucuoglu@marmara.edu.tr

**Keywords:** visual localization, mobile robot, text recognition, map

## Abstract

Robust autonomous navigation systems rely on mapping, locomotion, path planning, and localization factors. Localization, one of the most essential factors of navigation, is a crucial requirement for a mobile robot because it needs the capability to localize itself in the environment. Global Positioning Systems (GPSs) are commonly used for outdoor mobile robot localization tasks. However, various environmental circumstances, such as high-rise buildings and trees, affect GPS signal quality, which leads to reduced precision or complete signal blockage. This study proposes a visual-based localization system for outdoor mobile robots in crowded urban environments. The proposed system comprises three steps. The first step is to detect the text in urban areas using the “Efficient and Accurate Scene Text Detector (EAST)” algorithm. Then, EasyOCR was applied to the detected text for the recognition phase to extract text from images that were obtained from EAST. The results from text detection and recognition algorithms were enhanced by applying post-processing and word similarity algorithms. In the second step, once the text detection and recognition process is completed, the recognized word (label/tag) is sent to the Places API in order to return the recognized word’s coordinates that are passed within the specified radius. Parallely, points of interest (POI) data are collected for a defined area by a certain radius while the robot has an accurate internet connection. The proposed system was tested in three distinct urban areas by creating five scenarios under different lighting conditions, such as morning and evening, using the outdoor delivery robot utilized in this study. In the case studies, it has been shown that the proposed system provides a low error of around 4 m for localization tasks. Compared to existing works, the proposed system consistently outperforms all other approaches using just one sensor. The results indicate the efficacy of the proposed system for localization tasks in environments where GPS signals are limited or completely blocked.

## 1. Introduction

In recent years, outdoor mobile robots have become crucial due to their versatility and extensive range of applications. Outdoor mobile robots are employed for various tasks, including delivery [1], planting and harvesting in agriculture [2], security and surveillance [3], and maintenance of the supporting infrastructure [4]. Navigation is the most critical issue for mobile robots in accomplishing their assigned tasks successfully [5]. The success of navigation systems is based on factors such as location, mapping, path planning, and locomotion. Localization refers to determining the robot’s position based on its environment, a previous point, or a given map. It can be performed incrementally, where the position is tracked over time and changed by the robot’s motions, or globally, where the pose is computed just once based on preliminary observations [6]. Numerous localization techniques have recently been proposed and developed for various applications in indoor and outdoor environments. For outdoor environments, Global Positioning Systems (GPSs), a satellite-based navigation system, forms a crucial part of Global Navigation Satellite Systems (GNSSs), which are widely utilized methods in the literature in various outdoor applications to determine the precise locations of mobile robots.

**Figure 1:**
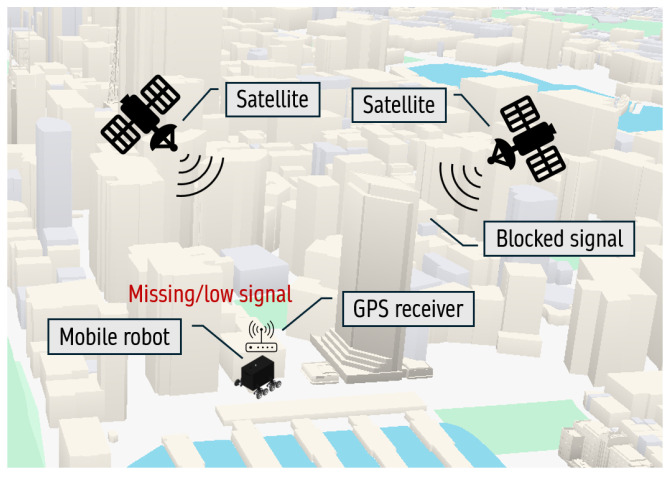
Problem illustration.

Despite its widespread implementation, localization through GPS might face some problems, such as accuracy problems or signal loss due to various environmental factors. Common reasons for accuracy or signal loss, which is illustrated in Figure 1, according to official US government information on GPS and related subjects [7] are (1) blockage of satellite signals by significant environmental factors such as skyscrapers, buildings, bridges, and trees; (2) reflection of signals from walls or buildings; and (3) both indoor and underground use. Depending on these factors, signal reception between the receiver and the satellite plays an important role. There are three forms of reception of GPS signal: line of sight (LOS), multipath, and non-line of sight (NLOS) [8]. In the LOS type, there are no obstructions or signal disturbances in the direct signal line between the satellite and the GPS receiver. This type of signal reception is ideal for obtaining the most reliable and accurate GPS signals. Conversely, in multipath reception types, the GPS receiver receives the direct LOS signals and the reflected signals from surrounding objects such as buildings, trees, and bridges. These reflected signals can negatively interfere with the accuracy of the location data received by the receiver. In the context of GPS signal reception types, the signal can be blocked or less reliable in an NLOS scenario, where the receiver can only receive the reflected signals. NLOS scenarios frequently occur in cities where towering structures surround a GPS receiver. Comprehending the various types of GPS signal reception is crucial to understanding the difficulties in achieving accuracy and dependability, especially in urban environments where the direct LOS connection between satellites and GPS receivers is blocked. The challenges in NLOS scenarios have an enormous effect on robotics, affecting the capability of robots to navigate, perform the given tasks, and move effectively in environments where direct GPS signal reception is blocked. Developing and implementing sensor fusion systems, such as adding more sensors, like cameras, lidars, or inertial measurement units, is necessary to overcome these challenges. For example, using camera sensors, environmental information can be sensed, such as street labels, store names, and other contextual information, which can later, with a few processes, obtain the robot’s actual position. Thus, it can significantly help overcome challenges associated with blocked GPS signals in urban areas.

Given the significant limitations of GPS signals in urban environments, such as signal blockage and accuracy issues caused by LOS, multipath, and NLOS scenarios, it is imperative to develop alternative systems that do not rely solely on GPS. Advanced localization methods, such as sensor fusion and leveraging environmental data, are essential to ensure reliable navigation and positioning for mobile robots, especially in challenging outdoor scenarios.

In urban areas, the perception of environmental information and scene texts, such as names of streets, stores, restaurants, etc., is a crucial feature for not only mobile robot navigation but also intelligent traffic systems, visual assistance, and other applications [9]. If adequately processed, such data can provide essential contextual cues for that environment or place. However, real-time text detection and recognition in urban areas pose significant challenges due to natural environmental factors such as lighting, obstructions, weather conditions, shooting angles, and considerable variability in scene characteristics in terms of text size, color, and background type [10]. Text detection, a prerequisite for text recognition, is a critical phase in extracting and understanding textual information. There are two types of methods for text detection in literature: traditional and deep learning-based methods. Conventional methods rely on manually designed features to capture the characteristics of scene text [11,12], whereas deep learning-based techniques extract features entirely from training data [13,14]. When text is accurately detected in real-time scenarios, it provides a clearer and more refined input to the Optical Character Recognition (OCR) system, resulting in higher recognition accuracy. OCR technology excels in recognizing documents with consistent background colors, basic fonts, and well-aligned text. However, the performance of scene text recognition, such as street names, store names, restaurant names, etc., is limited due to the complex backgrounds, distinctive and distorted fonts, uneven illumination, and color variations [15]. Obtaining and integrating this data type with other fields, such as location-based web mining, augmented reality, and geo-tagging, will facilitate various applications, including mobile robot localization.

Multi-sensor fusion systems significantly enhance navigation accuracy by addressing the limitations of single-sensor systems. These systems integrate various sensors, such as laser sensors, cameras, and sonar, to provide robust and reliable navigation solutions [16,17]. Sensor fusion enables robots to operate effectively in both structured and unstructured environments. Techniques like Space and Time Sensor Fusion (STSF) utilize temporal data sequences to improve measurement accuracy without requiring additional sensors [18]. Robots can achieve real-time obstacle avoidance by combining data from vision and sonar sensors, allowing them to navigate efficiently in unknown and dynamic environments [19,20]. However, implementing sensor fusion systems is complex and costly due to the need for integrating multiple sensors and developing sophisticated algorithms for effective data processing [18,20]. While sensor fusion significantly improves navigation, challenges remain in ensuring reliability and robustness, particularly in highly dynamic or poorly lit environments. Continued research is essential to address these limitations and enhance the adaptability of these systems [21].

**Figure 2:**
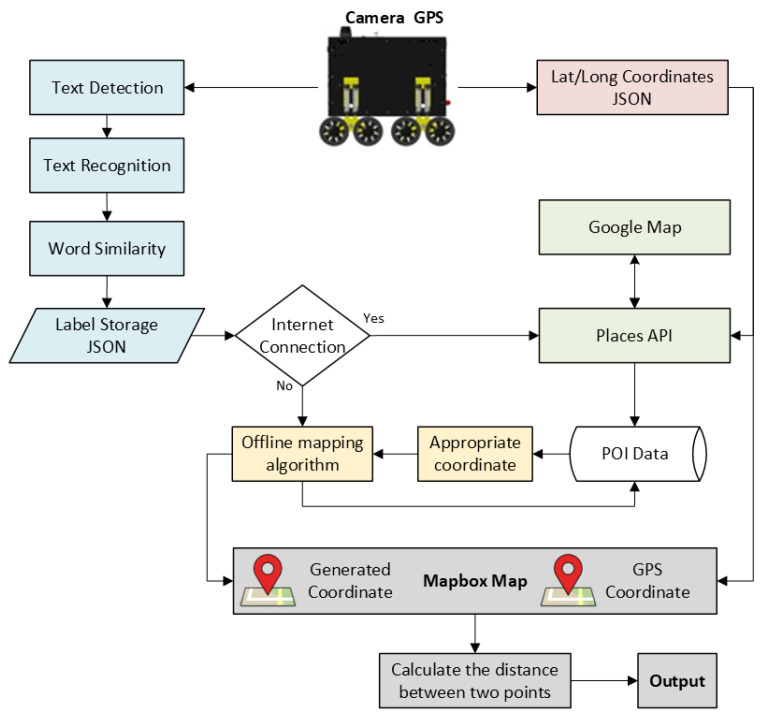
Flowchart of the proposed system.

Previous studies focused on localization in urban environments have been valuable in shedding light on effective technologies and solutions. However, they have certain limitations. Most previous studies in outdoor localization, particularly in scenarios where GPS signals are denied, commonly employ multiple sensors or sensor fusion techniques. Additionally, despite implementing diverse techniques, the challenge of high localization error persists in urban environments for robots. The primary objective of this study is to develop and implement a visual-based localization system for mobile robots in urban environments, particularly for areas where GPS signals are not accurate or totally denied. In addition, to represent the coordinates from the proposed system and GPS module, we developed a web-based map utilizing the Mapbox tool. The flowchart of the proposed system is demonstrated in Figure 2. Among the noteworthy contributions are:

- Optimize the EAST algorithm by utilizing a post-processing approach.
- Enhancement of the OCR algorithm by combining it with a word similarity algorithm for improved recognition accuracy and making it suitable for real-time applications.
- Develop a map that can operate offline and integrate it with the proposed system to display generated GPS coordinates in real-time.
- Obtaining a comprehensive and up-to-date point of interest (POI) database to achieve more accurate and reliable localization. Moreover, the POI data can be utilized for various applications in specific areas.
- Verify the proposed system’s reliable and simultaneous operation in real-time cases where GPS signals cannot be received or are weak in urban areas with high-rise buildings.
- The performance analysis of the proposed system was tested in real-time utilizing the eight-wheeled delivery robot by creating five different scenarios in different crowded urban environments such as crowded streets, pedestrian streets, and commercial districts.
- Analyzing the performance of the proposed system in different light conditions by performing experiments in both morning and evening hours.
- In contrast to prior research, which employed costly sensors or sensor fusion, in our study, localization was accomplished using only a basic webcam. This increases the usability of the proposed system.
- In contrast to previous studies that omitted the presentation of localization time for their systems, we showcase the localization timing of the proposed system.

This paper is organized as follows. Section 2 presents the related works on outdoor mobile robot navigation. Section 3 explains the methods of the overall system, while Section 4 presents validation of the proposed approach on the mobile robot for real-world applications. This paper is concluded in Section 5.

## 2. Related Works

Localization is an essential requirement that allows the robot to determine its location, as well as features such as map creation, optimal route planning, etc., [6]. In general, the localization can be accomplished with the aid of GPS signals, which provide a global position, or by onboard sensors. However, GPS localization reliability is insufficient, especially in urban environments where buildings may obstruct or reflect satellite signals. To overcome these limitations, many different approaches have been proposed to solve the localization problem. Encompassing approaches based on vision, light detection, range (LiDAR), inertial measurement unit (IMU), etc.

In [22], a visual localization method based on place recognition is introduced. This method realizes localization by recognizing recently visited places through the utilization of sequence-matching techniques. It operates by comparing query image sequences with a previously acquired image database. Moreover, a global GIST descriptor and a local binary feature CSLBP (center-symmetric local binary pattern) are combined to create a multi-feature to improve the matching accuracy. Finally, a Chi-square distance similarity measurement is employed for efficient sequence matching. The proposed system was evaluated on the Nordland dataset [23]. The dataset includes video footage capturing a 728 km long train ride between two cities in northern Norway. Precision and recall metrics are performed to evaluate the proposed method. The results show that the proposed system achieves more than 87% recall for spring–summer and spring–fall situations. The localization results also show that the suggested method can localize at least 60% of the test data. Experiments were conducted using a simulation; the paper does not include real-time localization experiments by the proposed system [22].

For accurate robot localization in challenging outdoor environments, [24] applied the Pozyx Algorithm, a hardware-based Ultra-Wideband system. The EKF was performed additionally. Thus, the output from the Pozyx algorithm was fed to EKF to fuse odometry with the Pozyx Range measurements. The experiments were carried out with AGROBv.16, a cost-effective outdoor robot. In [24], since the obtained GPS data were not accurate and dependable, the authors used a Laser Scan as the ground truth to analyze the results. From the experiments, it can be concluded that the proposed EKF implementation presents better results than the Pozyx algorithm. However, it requires careful tuning to ensure proper convergence.

In [25], a real-time CNN-based architecture was proposed, integrating data from low-cost sensors on a mobile robot with information derived from images captured by a single monocular camera. The system utilizes an EKF to execute precise relocalization of the robot in real-time. The proposed approach begins with the training of a CNN, which takes RGB images from the camera as input and applies regression for robot pose. Subsequently, the approach integrates the relocalization output obtained from the trained CNN into an EKF for robot localization. The system was tested in GPS-denied indoor and outdoor environments. The results show that the proposed CNN-EKF approach can generate accurate localization. However, the system faces difficulty providing accurate and simultaneous predictions for the robot’s location in environments characterized by repetitive scenes or visual content.

In [26], two approaches were proposed for localization: A Multi-Layer Perception Neural Network (MLPNN) for indoor localization and a sensor fusion-based approach for outdoor localization. In the sensor fusion system, the collected data from the inertial navigation system and GPS module are fused using recursive state estimation and a Kalman Filter. Then, the output from the Kalman Filter is combined with the odometer-based position data using a weighting scheme to estimate the robot’s location. The results show that the proposed scheme can provide location in environments where GPS signals are available or not. However, the authors do not provide the system’s accuracy or error rate. Additionally, the proposed system was evaluated in a simulation rather than a real environment.

To tackle the localization challenge, [27] proposed a localization system that fuses the data obtained from GPS, IMU, and visual odometry. In addition, the system involves EKF. The proposed system was evaluated in two outdoor environments. The results obtained by the system were compared with the raw data from the GPS module to measure the system’s robustness. Comparison results demonstrate that the proposed system exhibited an error rate of approximately 4 m, in contrast to the GPS module, which showed an error rate of about 79 m.

In [28], a low-cost approach was proposed for mobile navigation robots in indoor and outdoor environments. The system integrates data from multiple sensors, particularly low-cost visual and inertial sensors. For outdoor environments, an Extended Kalman Filter (EKF) was employed alongside GPS, wheel encoders, and a Reduced Inertial Sensor System (RISS) to estimate the robot’s position. Another EKF algorithm was introduced for indoor environments, utilizing a low-depth sensor called Microsoft Kinect Stream. Experimental results demonstrated that the proposed approach provides acceptable performance for real-time applications.

Based on deep learning and landmark detection, two methods were proposed to localize the mobile robot in outdoor environments [29]. The first proposed method was based on Faster Regional Convolutional Neural Network (Faster R-CNN) landmark detection in the acquired image [29], which obtains the robot’s location coordinates and compass orientation by utilizing detected landmarks. The second method performs a single convolutional neural network (CNN) to predict the location and compass orientation from the entire image. The experiments were conducted in two outdoor areas. The experimental results show that the Faster R-CNN average distance error was 28 m, while the distance error of the CNN model was around 70 m.

In [30], a visual localization approach was introduced, integrating depth and semantic information. The proposed approach employs semantic segmentation to capture a stable scene representation. It addresses variations in appearance between images caused by environmental changes by utilizing depth information obtained through depth prediction. The model was trained on the VKITTI 2 [31] and KITTI [32] datasets and subsequently evaluated on the Extended CMU Seasons and RobotCar Seasons datasets. The experimental results demonstrate that the proposed method exhibits remarkable performance in visual localization under various conditions, including weather, vegetation, regional environment, and illumination, based on evaluations conducted on the Extended CMU Seasons and RobotCar Seasons datasets.

In [33], a global image descriptor was introduced to address the challenges of image-based localization in demanding scenarios such as cross-season, cross-weather, and day–night conditions. The proposed descriptor can handle visual changes between images by learning the scene’s geometry. The strength of the proposed method lies in the fact that it only requires geometric information during the learning process. The proposed method was tested on the Oxford Robotcar public dataset [34] and the CMU Visual localization dataset [35]. The proposed descriptor demonstrates remarkable performance in challenging cross-season localization scenarios, making it a valuable solution for long-term place recognition. Moreover, promising results are achieved in the context of night-to-day image retrieval.

In [36], an end-to-end DL-based visual localization algorithm was proposed. Pre-processing tasks, including cropping, averaging, and timestamp alignment, are executed on datasets to minimize computational cost and time. Then, the processed dataset was fed to the proposed CNN-RNN-based model to identify the most impactful features for matching. Finally, the system predicts and provides output for the robot’s current 3D translation and 4D angle information, thereby realizing a fully integrated end-to-end localization system. The proposed method was evaluated using three distinct datasets: the Cambridge Landmarks outdoor dataset [37], the Microsoft 7-Scenes indoor dataset [38], and the TUM Handheld SLAM dataset [39]. During testing in the Cambridge Landmarks for outdoor environments, the proposed method exhibited promising results for localization. Nevertheless, there is currently a deficiency in implementing the proposed model for real-time applications in mobile robots.

In [40], an innovative method for long-term robot localization using solely monocular image data is proposed. The proposed approach involves a unique data association technique to match incoming image streams with a stored image sequence in a database. Leveraging network flows, the method enhances localization performance by incorporating sequential information and maintaining multiple trajectory hypotheses simultaneously. Image comparison relies on a semi-dense description employing a histogram of oriented gradients’ features and global descriptors from deep convolutional neural networks trained on ImageNet, ensuring robust localization. The proposed approach demonstrates respectable performance in localization tasks through comprehensive evaluations across diverse datasets. The evaluation was carried out on datasets that were collected by driving through a city with a camera-equipped car during different seasons, including summer and winter.

## 3. Methodology

This section explains the processes of text detection, recognition, word correction, web-based coordinate generation, map development, overall system integration, and the mobile robot platform in detail.

Firstly, hardware modifications were carried out on the mobile robot to align it with the objectives of this study and prepare it for experimental scenarios. After reviewing the literature and conducting experiments on text detection and recognition in urban areas, we implemented the EAST algorithm for text detection and EasyOCR for text recognition. To enhance the performance of the EAST algorithm, we applied Non-Maximum Suppression (NMS), which eliminates redundant bounding boxes and ensures more accurate text detection. Although EasyOCR is commonly used for documents, it has limitations in real-time applications, such as missing or incorrectly recognized characters, making it insufficient for standalone use. To address these issues, the Sequence Matcher algorithm was incorporated to correct errors in character recognition. This step significantly improved the performance of EasyOCR in real-time text recognition tasks. Another challenge in text recognition involved handling different fonts and variations in uppercase and lowercase letters. All POI data, mainly business names, are stored in lowercase for consistency. However, text recognition outputs often include mixed cases. All recognized text is converted to lowercase using the toLowerCase() function to ensure accurate searches and coordinate retrieval from the JSON file. To localize the mobile robot in scenarios where GPS signals are weak or unavailable, we obtained POI data for specific areas using the NS API. The retrieved POI data was stored in a JSON file. Once a business name is recognized, the algorithm searches the JSON file and retrieves the corresponding POI data. The Haversine formula is applied to calculate the nearest coordinates within a defined radius, returning the business name and its associated coordinates. These generated coordinates are displayed on a map as blue points, while coordinates obtained from the GPS module are shown as red points. We developed a web-based map using Mapbox to visualize and track the coordinates. The system-generated coordinates and the GPS module coordinates are displayed on the map for comparison. To evaluate the accuracy of the proposed system, the distance between the system-generated coordinates and the GPS coordinates is calculated using the Haversine formula. This comparison provides a visual and quantitative measure of the system’s performance, with GPS coordinates displayed as red points and system-generated coordinates as blue points.

### 3.1. Text Detection and Recognition

This part will explain the used sensors, text detection, post-processing, text recognition, and word correction processes.

#### 3.1.1. Sensors

This study employs two sensors: a camera for visual perception and a GPS module for obtaining reference coordinates. We utilized the Logitech C922 [41] for the camera. This camera captures videos at 1080p High Definition (HD) resolution with a 78-degree field of view and a frame rate of 30 frames per second. The Logitech C922 was selected for its numerous advantages, including high resolution, autofocus capability, lightweight design, and affordability.

For GPS modules, we tested the Quectel L80 and the Ublox Neo-M8 [42]. To determine the most suitable module for the experiment, we measured their errors as follows: First, the actual coordinates of the test location were obtained from Google Maps. Then, the coordinates provided by each GPS module were recorded. Finally, the error for each module was calculated using the “measure distance” tool in Google Maps to compare the recorded coordinates with the actual location. The results indicated that the Ublox Neo-M8 GPS module had a lower error. Consequently, the Ublox Neo-M8 was chosen for this study.

#### 3.1.2. Scene Detection

**Figure 3:**
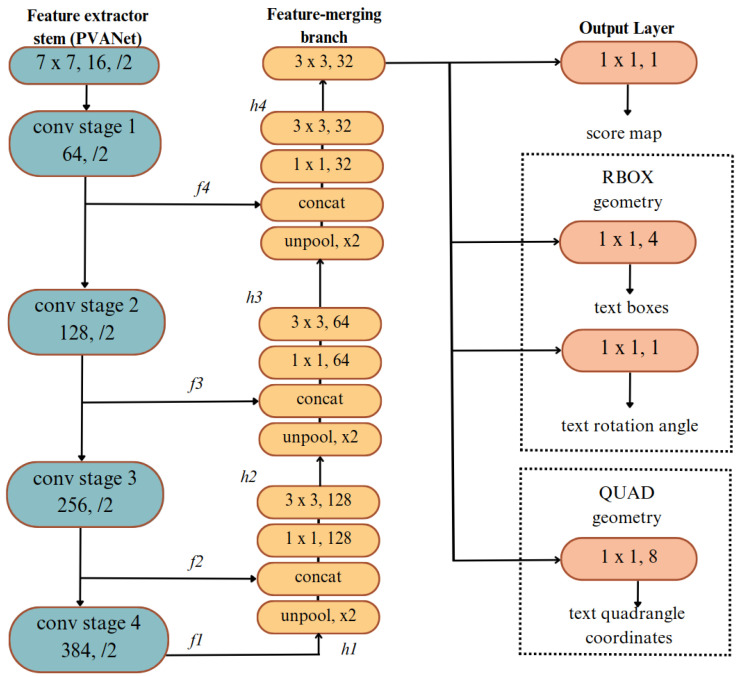
Architecture of EAST algorithm.

Scene detection algorithms use image processing and computer vision techniques to detect text in complex scenes with high accuracy and efficiency. These algorithms facilitate detection and recognition in numerous fields, including security systems, logo and signage identification, traffic signal interpretation, etc. Based on the findings in [43], the EAST algorithm outperformed other methods in terms of speed and accuracy, leading to its selection for use in this study. The EAST [44] algorithm, introduced in 2017, was designed to overcome the challenges of scene detection in complex environments. It utilizes only one neural network to predict words or lines of text by passing the need for candidate aggregation and word segmentation. The model architecture is demonstrated in Figure 3.

EAST employs a convolutional neural network (CNN) as its backbone to extract feature maps from input images. The feature maps from various layers of the CNN backbone are merged to create a more detailed and spatially precise feature map. This is achieved through multiple branches and concatenation, which enhance the model’s ability to detect text effectively in complex scenes. In the following equation:

(1) FusedFeature=Concat(F1,F2,…,FN)

(F1,F2,…,FN) are feature maps from different layers of the backbone network. The model has two key output branches: score map and geometry map. The score map predicts the probability of each pixel belonging to text or non-text. It outputs a score map; the model has two key output branches: the score map and the geometry map. The score map estimates the likelihood of each pixel being part of text or non-text regions. The following equation represents this:

(2) $$S\left(x,y\right)=\sigma \left(f_{\mathrm{score}}(F_{\mathrm{fused}}\left(x,y\right)\right))$$

where $(F_{\mathrm{fused}}\left(x,y\right)$ represents the fused feature map at the position “(x,y)” and $f_{\mathrm{score}}$ applies a transformation to predict the score, followed by a sigmoid activation function σ to produce the final probability. The geometry map predicts the geometry of the text boxes using two formats: RBOX and QUAD. The RBOX format is defined by 5 parameters, with 4 representing the corners of a rotated rectangle and 1 representing the rotation angle, while the QUAD format uses 8 parameters to describe the four vertices of a quadrilateral. The output of this branch is a geometry map represented by the following equation:

(3) $$G\left(x,y\right)=\left(f_{\mathrm{geo}}(F_{\mathrm{fused}}\left(x,y\right)\right))$$

where $f_{\mathrm{geo}}$ is a convolutional layer that outputs geometry information.

#### 3.1.3. Post Processing

After the EAST model generated the score map and geometry map, we applied Non-Maximum Suppression (NMS) to remove redundant overlapping boxes, retaining only the most confident ones. The process begins by sorting all predicted boxes based on their confidence scores, selecting the box with the highest score, and suppressing other boxes that overlap significantly with it based on the Intersection over Union (IoU) metric. The IoU between two boxes, A and B, is calculated using the following equation:

(4) $$\mathrm{IoU}\left(A,B\right)=\frac{\left|A\cap B\right|}{\left|A\cup B\right|}$$

where |A∩B| represents the area of overlap between the two boxes and |A∪B| represents the total area covered by both boxes. Boxes with an “IoU” above a predefined threshold are suppressed.

#### 3.1.4. Optical Character Recognition

**Figure 4:**
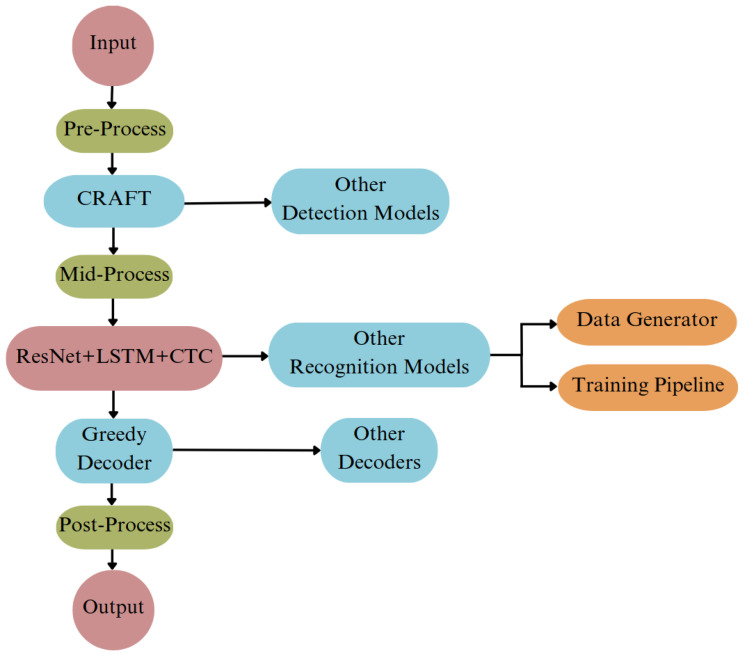
Architecture of EASY OCR algorithm.

Easy OCR is a highly efficient OCR library that supports over 80 languages, developed using Python and PyTorch. It employs a Convolutional Recurrent Neural Network (CRNN) [45] for text recognition, consisting of three main components: ResNet [46] for feature extraction, LSTM [47] for sequence labeling, and Connectionist Temporal Classification (CTC) [48] decoding. The workflow of Easy OCR is illustrated in Figure 4. This engine is particularly effective due to the advanced pre-processing steps included in its pipeline, making it one of the top OCR solutions available. Easy OCR was reconfigured to recognize Turkish text for our work, as our dataset is in Turkish. Once the text is detected and enclosed within a bounding box, it is processed by the OCR engine to recognize the text in the environment. The Easy OCR was used due to its performance, which is presented in [49].

#### 3.1.5. Sequence Matcher

Sequence Matcher (SM) [50] is a class of the “difflib” module used to compare the similarity of two given strings. The Ratcliff/Obershelp algorithm [51] is run in the background. After comparing the two given strings, the algorithm returns a score between 0 and 1. After comparing two strings, if the obtained score is greater than 0.7, it will be regarded as a keyword and stored as an actual word or label. The equation of the algorithm is as follows:

(5) $$D_{ro}=\frac{2*K_{m}}{\left|S1||S2\right|}$$

where $K_{m}$ represents the number of the same characters in sequence, whereas |S1| and |S2| give the corresponding length for each of these two strings.

### 3.2. Localization Based Web Mining

This part presents a detailed description of generating coordinates for localization tasks. In addition, the creation of the web-based map was explained. Furthermore, the developed algorithm for the overall system, encompassing various methods, is presented. Finally, the workflow of the proposed system is summarized before delving into the experiments.

#### 3.2.1. Google Places Data

Google Places is a comprehensive service provided by Google that offers detailed information about places and locations all around the world. It includes an extensive database with geographic data, business listings, and points of interest (POI). POI data define digital representations of specific locations or areas that interest some population segments [52]. POI data cover various types of locations, such as restaurants, markets, stores, grocery stores, schools, hospitals, and so on. POI data are pivotal for robotic localization, enabling them to navigate more efficiently for given tasks, particularly when GPS signals are limited or blocked. The Places API is crucial in providing necessary POI data for various locations. The Google Places extensive data can be accessed utilizing the Google Places API [53], which essentially serves as a search function. There are four search techniques available for locations:

- **NS** provides a list of nearby places based on a given location; input location data type should be in terms of latitude and longitude coordinates.
- **Text Search** returns a compilation of locations in the region based on a search string, for example, “Spaghetti”.
- **Place Details** returns more thorough details about a certain location, including user reviews.
- **Place Photo** enables the retrieval of photos associated with a specific place.

The Nearby Search (NS) [54] is a crucial search technique for location-based services. It enables users to find POIs near a specified geographic location. Developers can customize search queries based on various parameters such as keywords, language, open now, rank by, and type. However, two fundamental parameters are required for the NS function: location and radius. Users should define a central “location” by providing latitude and longitude coordinates, which will serve as the epicenter of the search. The “radius” parameter defines the region where the NS will be conducted. It is a virtual boundary, limiting the search’s range to a particular distance. Utilizing the “keywords” parameter is crucial in enhancing the search results. Searching by providing “keywords” or phrases as parameters is important for finding specific types of businesses, services, or any POI. The response from an NS provides extensive information on locations within a specified geographic area. These data usually include each place’s name, address, and geographical coordinates, allowing developers to present a dynamic and rich exploration of their environment.

In this paper, we utilized the NS option with JavaScript programming language. In accordance with the scope of the study, it was intended to extract POI data from a certain region and save it to a file with a “.json” extension by utilizing NS. To employ the NS, firstly, we feed the coordinates from the GPS module placed in the robot as a location parameter. As they represent the robot’s actual position, the given coordinates will serve as the epicenter of the search. Secondly, we define a parameter “keyword” to specify the business names. This keyword parameter enables us to retrieve more precise information about the businesses in the region. After providing the location and keyword parameters, the last step is defining a radius to search for businesses in a specific region. We set the radius between 1.500 to 2.000 m depending on the scenario. After applying all the steps, we collect POI data for particular regions where the experiments will be performed regarding business name, coordinates in latitude and longitude form, business status, rating of the business, and so on. All the collected data are stored in a JSON file for further use in the final system. In the final system, where localization is the main goal, the crucial elements of each obtained POI data are the business names and their corresponding coordinates. As can be seen, the data include location in the form of lat and long, business name, business status, rating, address, type of place, and so on.

#### 3.2.2. Mapbox

Mapbox [55] is a web platform that offers powerful tools and services for incorporating interactive and customizable maps. Mapbox provides various mapping services, including navigation, geocoding, spatial data analysis, etc. Mapbox creates compelling visual and dynamic maps by combining raster and vector tiles. Mapbox offers a variety of map styles, and developers can choose from pre-designed styles or make their own using Mapbox Studio. Additionally, the platform provides software development kits (SDKs) for other programming languages, opening it up to a large developer community. On the other hand, Mapbox offers a variety of map styles that developers can utilize to customize the appearance of their maps. Different map styles can be chosen depending on the application’s purpose, audience, and design preferences. Mapbox has offline map functionality. Thus, developers can create custom offline maps without requiring an internet connection. This is especially helpful when users might not always have internet connectivity, such as in remote areas or with applications requiring reliable offline functionality. In this paper, a Mapbox-based web map was developed to demonstrate the coordinates obtained from the GPS module and those generated by the proposed system. The development of the map involves the incorporation of Mapbox GL JS, a powerful JavaScript library designed for creating interactive and customizable maps. Utilizing Mapbox GL JS enables us to render map data from Mapbox Vector Tiles, employing the Mapbox Style specification and hardware-accelerated graphics (WebGL). As a map style, we employed ’streets-v12’ [56], which is Mapbox’s predefined style, offering a detailed representation of streets, landmarks, and geographical elements. In addition, Google Places was integrated with the map for utilizing Google Maps services. While developing the map, the map’s initial view is centered at defined coordinates, with a zoom level set to 6. The map has been configured to operate offline without an internet connection. Once the map has been created, we define two points on the map. The first point, denoted in red, represents the coordinates obtained from the GPS module, while the blue point represents the coordinates generated from the proposed system. In the final system, as we will receive coordinates sequentially from the GPS module and proposed system, the respective points will continuously update their location on the map.

#### 3.2.3. Haversine

The Haversine Formula is a mathematical equation based on trigonometric principles used to calculate the distance between two points on the surface of a sphere, often applied to measure the great-circle distance between two locations on the Earth. The initial tabulation of Haversines was introduced by Andrew in 1805. However, Inman officially coined the term “Haversine” in 1835 [57]. The Haversine formula represents a specific instance of the law of Haversines, establishing connections between the sides and angles of spherical triangles. The Haversine distance [58] measures the great-circle distance in kilometers between two points on a sphere. The formula is defined as follows:

(6) $$a=\sin^{2}\left(\frac{\Delta \phi }{2}\right)+\cos\left(\phi_{1}\right)\cdot \cos\left(\phi_{2}\right)\cdot \sin^{2}\left(\frac{\Delta \lambda }{2}\right)$$

where $\phi_{1}$ and $\phi_{2}$ are the latitude values at the two specified coordinates, Δϕ is change in latitude and Δλ is change in longitude.

(7) $$c=2\cdot atan2\left(\sqrt{a},\sqrt{1-a}\right)$$

The center angle between two points is determined by Equation (7).

(8) d=R·c

The shortest distance is determined by multiplying the value of *R* by *c*, where *R* corresponds to the Earth’s radius. Considering previous research in the field, specifically regarding distance computation between points and subsequent path length determination, we will employ the Haversine distance instead of the Euclidean in this paper. In this case, we implemented the Haversine formula to calculate distances between GPS module coordinates and generated coordinates. Furthermore, we integrated it into our developed algorithm to obtain the most relevant coordinate corresponding to a given label.

The Haversine formula is based on trigonometric functions, which normally require radians rather than degrees. Before applying the Haversine Formula to measure the distance between two points, we convert the latitude and longitude coordinates from degrees to radians with the following equation:

(9) $$lat_{rad}=\frac{lat\cdot \pi }{180}$$

(10) $$lon_{rad}=\frac{lon\cdot \pi }{180}$$

After converting the degrees to radians, we apply the Haversine Formula to calculate the distance between two points. The output distance is typically in kilometers, reflecting the Earth’s radius *R* used in the Formula (8), which is approximately 6371 km. Finally, to obtain the distance in meters, we convert from kilometers to meters as follows meters=kilometers·1000.

### 3.3. Mobile Robot Platform

The experiments were conducted using an 8-wheeled mobile robot developed in [59]. This robot features eight rubber wheels, providing the ability to climb pavements, and is powered by eight brushed DC motors. High gear-ratio motors were selected to ensure reliable performance on uneven outdoor terrains. The motors are controlled via an Arduino Mega 2560, a microcontroller board based on the Atmega2560.

In the robot’s default setup, the camera’s position was misaligned with the target views, which could adversely affect the performance of the text detection and recognition algorithms. To address this, a platform was designed and mounted on top of the robot, aligning the camera with the target views.

During the experiments in [49], vibrations caused by the rubber wheels and uneven ground resulted in blurred video footage, negatively impacting the algorithms’ performance. To mitigate this issue, a gimbal equipped with a Logitech camera was installed on top of the platform, stabilizing the camera. After these modifications, the camera was positioned 1.08 m above the ground.

**Figure 5:**
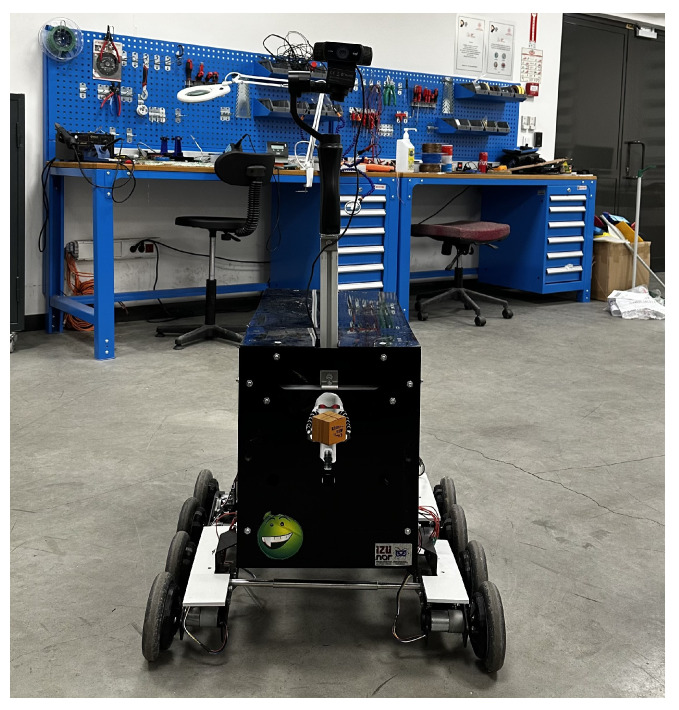
A view of the robotic platform from the laboratory environment.

The robot was equipped with a laptop running a 64-bit Ubuntu 20.04 Linux operating system. The laptop featured an Intel i5 processor, 8 GB of RAM, and an Nvidia GeForce 1680 graphics card. GPU functionalities were utilized by installing CUDA and CuDNN libraries. The final appearance of the robot, following these modifications, is illustrated in Figure 5.

## 4. Case Studies

This section will present the experiments conducted using the proposed system and compare the results with existing works.

**Figure 6:**
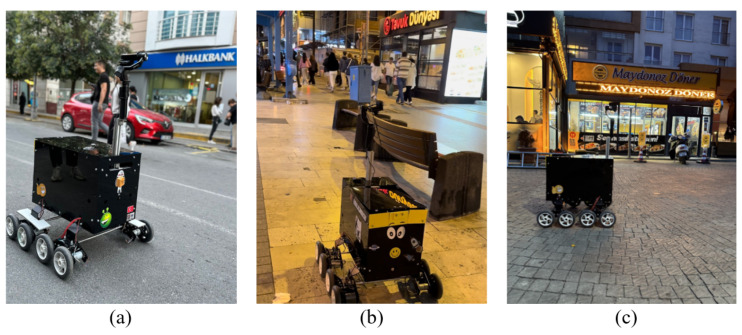
Robot samples for each Area. (**a**) Test Area 1, (**b**) Test Area 2, (**c**) Test Area 3.

In order to evaluate the accuracy of the proposed system and determine its capability to localize the robot in cases where GPS signals are lost, two types of scenarios were performed: (1) Obtaining coordinates from the GPS module and proposed system and (2) Obtaining coordinates from the proposed system. The coordinates from the GPS module are demonstrated on the map with a red point, while the coordinates from the proposed system are demonstrated with a blue point. The experiments were carried out on three distinct urban areas: Crowded Street (Area 1), Pedestrian Street (Area 2), and Commercial District Street (Area 3). In Figure 6, the robot samples for each area are illustrated.

A crowded street operates as a one-way street for traffic movements, experiencing particularly heavy traffic, especially during the morning hours. The most common types of businesses along the street are banks and stores. The street surface includes only asphalt. The street with pedestrians operates as a thoroughfare exclusive to pedestrians and is closed to traffic. Commercial establishments, including stores and food stores, characterize the street. The street surface consists of tiles as well as asphalt in some regions. The crowded street is open to two-way traffic and has pavement for pedestrians’ use. The street includes a variety of businesses, such as coffee shops, markets, restaurants, and so on. The surface varies from region to region, including asphalt, concrete, and cobblestone. Considering the area in which the robot can move within the streets, the test area length of the experiments varies. The robot was generally operated at an approximate velocity of 5 km/h. However, owing to traffic jams and the presence of pedestrians in the experimental area, there were instances where it was necessary to either stop or reduce the velocity.

### 4.1. Case Study 1: Localization in Crowded Urban Streets

**Figure 7:**
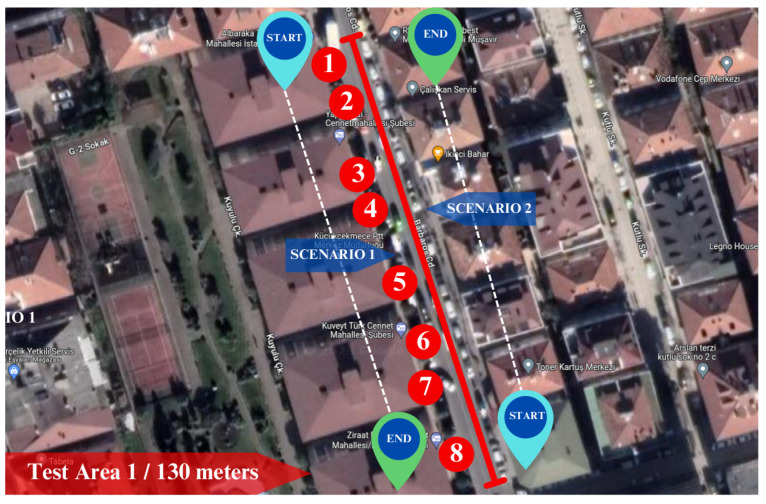
Start and end points of the robot for Test Area 1.

The first experiment was conducted in a crowded urban street in test Area 1. As illustrated in Figure 7, start and end points were defined for the motion of the mobile robot. The length of the test Area 1 is around 130 m. The street of test Area 1 is surrounded by five or six-storey buildings. The surface of the street is asphalt. Thus, utilizing the gimble on the robot can avoid the vibrations caused by the surface, resulting in a good video stream. The numbers in Figure 7 represent the businesses that are referred to in Table 1. There are eight businesses, six of which are banks, one store, and one cargo store. Each business consists of different signboards with its name with various types of fonts and backgrounds.

**Scenario 1**

In this scenario, coordinates from the GPS module and generated coordinates are obtained to evaluate the performance of the proposed system. The experiment was conducted in the late afternoon; it began at 5:55 PM and ended at 6:01 PM. The robot’s starting point is at the beginning of the business building marked with number one, and its ending point is at the building denoted by number eight in Figure 7.

**Figure 8:**
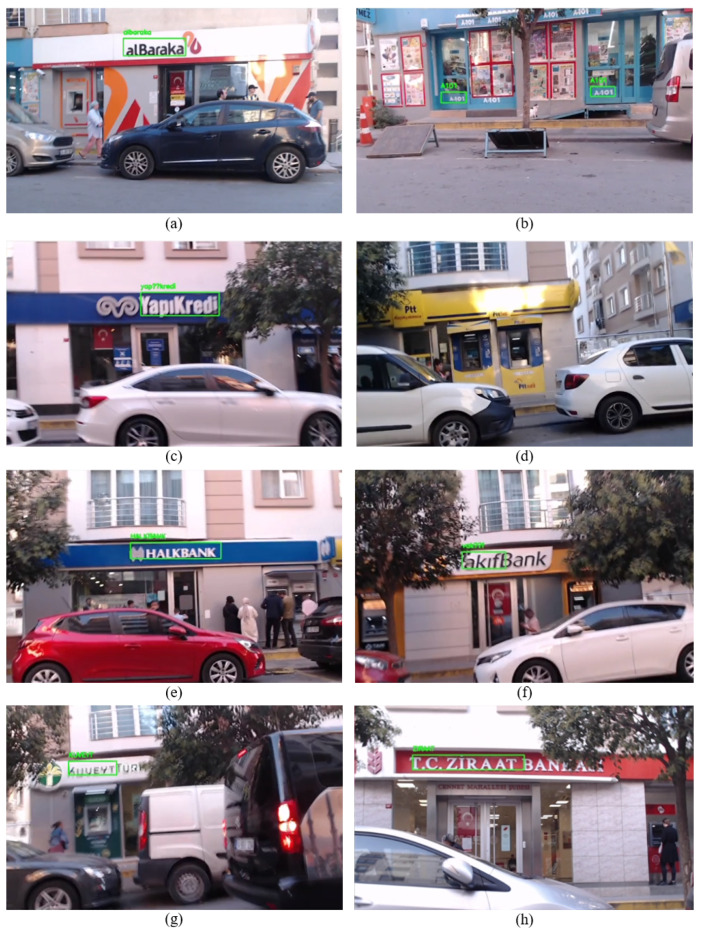
Detected and recognized business names by the proposed system in Scenario 1. (**a**) “alBaraka”, (**b**) “A-101”, (**c**) “YapıKredi”, (**d**) “PTT”, (**e**) “HALKBANK”, (**f**) “VakıfBank”, (**g**) “KUVEYTTÜRK”, (**h**) “Ziraat Bankası”.

As the mobile robot started moving, text detection and recognition were performed. In Figure 8, from the (a) to the (h), the texts detected and recognized in real-time by the enhanced system are demonstrated, respectively, as mentioned in Table 1. When the real-time video stream was handled, it was seen that the enhanced system could easily identify the label “alBaraka” from a variety of angles, even from the angles where sunlight exists, as can be seen in Figure 8 under option (a). As can be seen under option (b), the business, namely “A-101”, does not have a signboard where its name is mentioned. The algorithm successfully recognized the business’s name from the small label on the door. It was comparatively easier for the algorithm to identify the business name in options (c) and (e) due to the same background color and font used for businesses’ signboards. In particular, “HALKBANK” was detected more often during the stream because of its name’s capitalization. The enhanced system could not recognize the business name “Ptt”. As can be seen from option (d), the characters of the business name are adjacent to each other. Therefore, the algorithm can not separate the characters, resulting in the interpretation of the name as a single character rather than individual characters. It has been noted that the algorithm performs more efficiently when handling business names (signboards) with dark color tones and white backgrounds. The algorithm frequently recognized the businesses “Vakıfbank” and “KuveyTürk” even when blurriness occurs in the video stream, as can be seen under options (f) and (g). The last one, “Ziraat” was easily recognized from various angles due to its capital characters. In the overall process of detecting and recognizing text on the street in the first scenario, the enhanced system effectively recognized all business names along the route except one name. This success was achieved under various challenges such as lighting variations, image blurring due to the vibrations, and traffic jams.

In Table 2, recognized business names (labels) with EasyOCR and corrected labels by Sequence Matcher with the similarity rate are presented. The Word Similarity Rate in Table 2 reflects the similarity between the word recognized by the OCR algorithm and the actual word before applying the Sequence Matcher. In scenario 1, EasyOCR recognized the business names “alBaraka”, “A-101”, “YapıKredi” and “Vakıf” correctly. Thus, the similarity rate is 100%. The algorithm recognized the labels “HALKBANK”, “KUVEYTTÜRK” and “Ziraat” as missing one or two characters. Therefore, the similarity rate varies. The reason for missing one or two characters is that some look similar. For example, the algorithm recognized “HALKBANK” as “HAIKBANK”; here, the similar look of the characters “I” and “L” negatively affects the EasyOCR algorithm. Finally, as mentioned above, the algorithm could not recognize the label “Ptt”. Therefore, it is demonstrated in the table with a dash (-). The obtained results show that utilizing the Sequence Matcher algorithm enhances the text detection and recognition system in Scenario 1.

Table 3 compares the GPS module coordinates and generated coordinates regarding distance error. In this context, distance error refers to the difference between the GPS module coordinate and the generated coordinate. In addition, the precision of the generated coordinate for each business was verified on Google Maps. We take GPS and generate coordinates for each business when the robot is in front of the business. When Table 3 is examined, it can be seen that the proposed system generated coordinates with 1.23 m error when the robot was positioned in front of the business “YapıKredi”. This signifies the lowest error rate. On the other hand, the highest error rate occurred when the robot was positioned near business “alBaraka” with a 6 m error rate. When the robot approached the businesses “A101”, “HALKBANK”, “VakıfBank”, “KUVEYTÜRK” and “Ziraat”, the error rates were measured as 3.27, 3.24, 5.67, 6.49, and 5.43 m, respectively. Consequently, the average error of the generated coordinates with respect to the coordinates of the GPS module is 4.48 m. This shows the robustness of the proposed system, affirming its capability to uphold stability and performance in urban environments.

**Figure 9:**
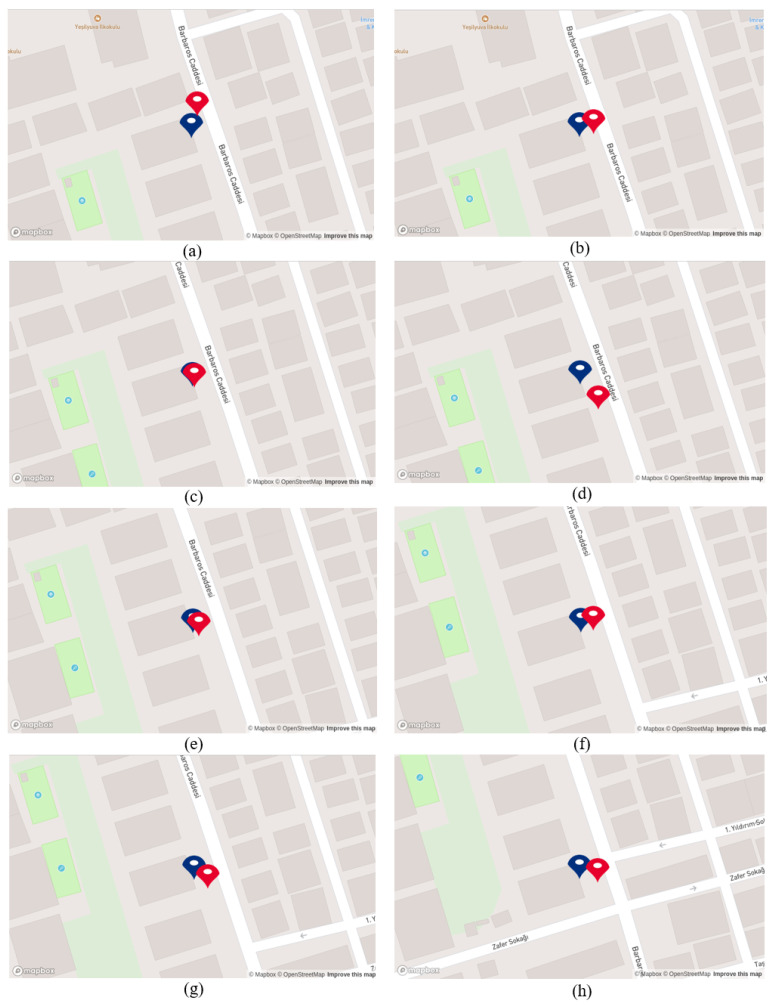
Representation of GPS coordinates and generated coordinates on map in Scenario 1. (**a**) “alBaraka”, (**b**) “A-101”, (**c**) “YapıKredi”, (**d**) “PTT”, (**e**) “HALKBANK”, (**f**) “VakıfBank”, (**g**) “KUVEYTTÜRK”, (**h**) “Ziraat Bankası”.

The proposed system generates coordinates once the business names are detected and correctly recognized. As illustrated in Figure 9, the generated coordinates and the coordinates obtained from the GPS module are demonstrated on the developed map. Note that the coordinates obtained from the GPS module are represented with red points, while generated coordinates from the proposed system are demonstrated with blue points. As the mobile robot moves, the red point on the map updates its location according to the coordinates from the GPS module, while the blue point is updated as the robot recognizes the business names and the system generates coordinates. When Figure 9 is examined from option (a) to (h), it can be seen that the blue point updates itself in synchronization with the red point as mobile robots come near the businesses and recognize the business names. Except in option (h), where the label “Ptt” was not recognized, the system could not generate a coordinate and update the blue point. The effective updating of the blue point on the map along the 130 m demonstrates the success of the suggested system.

**Scenario 2**

This scenario was carried out in the morning hours, under sunny weather conditions. The experiment started at 09:06 AM and ended at 09:11 AM. Despite dozens of attempts from various locations on the street, we could not get a signal from the GPS module due to environmental factors. Hence, in this scenario, while the mobile robot moves, it only updates its location on the map with the coordinates obtained from the proposed system. To measure the difference in the endpoint of the scenario, we manually define an end coordinate for the red point. Consequently, the robot will update its position based on the proposed system. At the end of the scenario, we will measure the distance between the manually defined coordinate and the robot’s position. The robot’s starting and end points are in contrast to scenario 1. In this case, the robot starting point is the business marked with the number eight, and the endpoint is the business denoted with the number one in Figure 7.

**Figure 10:**
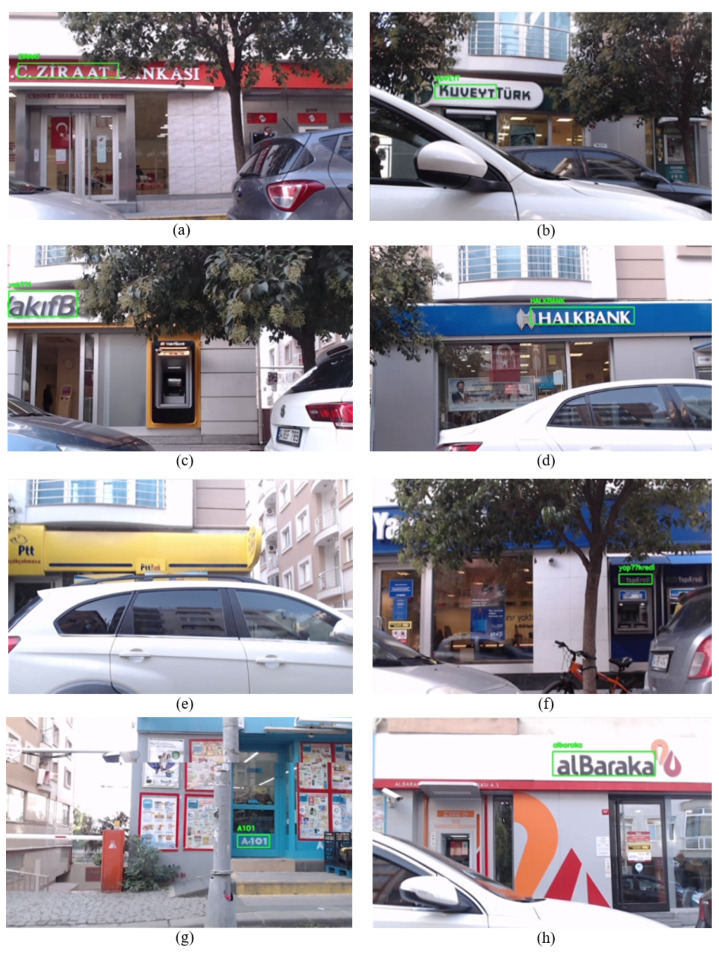
Detected and recognized business names by the proposed system in Scenario 2. (**a**) “Ziraat Bankası”, (**b**) “KUVEYTTÜRK”, (**c**) “VakıfBank”, (**d**) “HALKBANK”, (**e**) “PTT”, (**f**) “YapıKredi”, (**g**) “A-101”, (**h**) “alBaraka”.

Due to the traffic jam, in contrast to scenario 1, we drove the mobile robot closer to the businesses in this scenario. As the mobile robots move, the detected and recognized business names are illustrated in Figure 10 sequentially based on their locations in the street. As depicted in Figure 10, from option (a) to (h), the enhanced system successfully recognized each business name except “Ptt”. When the real-time video stream was handled, it was seen that even if the weather is sunny and there are good lighting conditions, the camera angle negatively affects the system’s detection and recognition. Due to the business names being too high concerning the camera angle, the improved system encountered challenges in recognizing the name “VakıfBank”; thereby, it recognizes the related name in the last phase of the video stream, as can be seen in Figure 10 under option (c). In addition to the camera angle issue, another notable challenge in Scenario 2 was the presence of trees and their shadows, which partially obscured parts of the signboards or created uneven lighting conditions. For instance, in Figure 10, under options (b), (c), and (f), tree branches and leaves blocked portions of the signboards, making it difficult for the detection algorithm to accurately identify the text. Environmental factors such as moving vehicles introduced blurriness on the video stream in some cases, such as in option (e). Despite these challenges, the rest of the business names were easily recognized multiple times from different angles, as seen under options (a), (b), (d), (f), (g), and (h), due to the Sequence Matcher.

In Table 4, it can be seen that EasyOCR, except for business names “A-101” and “alBaraka”, encounters challenges in recognizing business names. The EasyOCR algorithm recognizes the “Ziraat” as “iraa”, “KUVEYT” as “KUUEYT”, and “Vakıf” as “akıfB”, “HALKBANK” as “IHALKBA”, “YapıKredi” as “TapıKredi”. This is caused by the camera angle and the traffic sequence, making it blurry. To overcome this limitation, we integrated the OCR algorithm with the Sequence Matcher, which allowed us to correct misrecognized words and significantly improve accuracy, reaching from 80% to 100%. This low rate (around 80%) shows that the OCR algorithm alone may not always achieve high accuracy in real-time, particularly under challenging conditions. This integration enhances the real-time usability and reliability of the text recognition process. As a result, each business name was correctly recognized for the further process.

When Table 5 is evaluated, it can be seen that as the mobile robot reaches the last business, the distance difference with the red point decreases to 7.15 m. When the distance difference was evaluated for each business, it can be seen that the distance difference decreased simultaneously, which reveals that the proposed system creates smooth coordinates.

**Figure 11:**
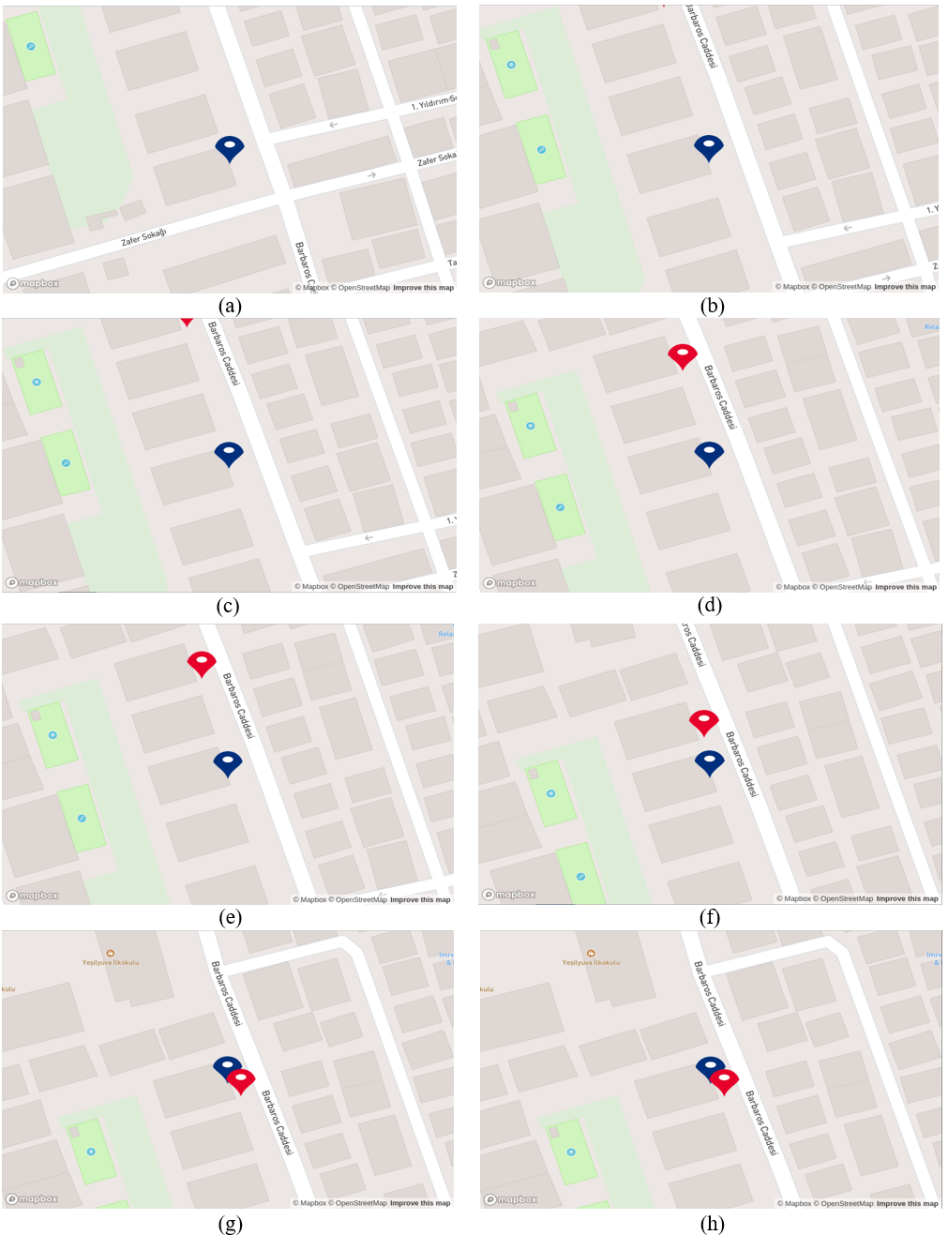
Representation of GPS coordinates and generated coordinates on map in Scenario 2. (**a**) “Ziraat Bankası”, (**b**) “KUVEYTTÜRK”, (**c**) “VakıfBank”, (**d**) “HALKBANK”, (**e**) “PTT”, (**f**) “YapıKredi”, (**g**) “A-101”, (**h**) “alBaraka”.

Upon correctly recognizing the business names, the proposed system generates coordinates. As was mentioned before, we define constant coordinates for the red point due to the GPS signal blockage. Consequently, while the mobile robot is in motion and the proposed system operates in the background, the blue point continuously updates its position, as depicted in Figure 11. When the mobile robot reaches the end of the route, the blue point comes closer to the red point. Figure 11 reveals that as the mobile robot reaches the end of the route, the blue point approaches the red point.

#### 4.1.1. Case Study 2: Localization in Pedestrian Streets

**Figure 12:**
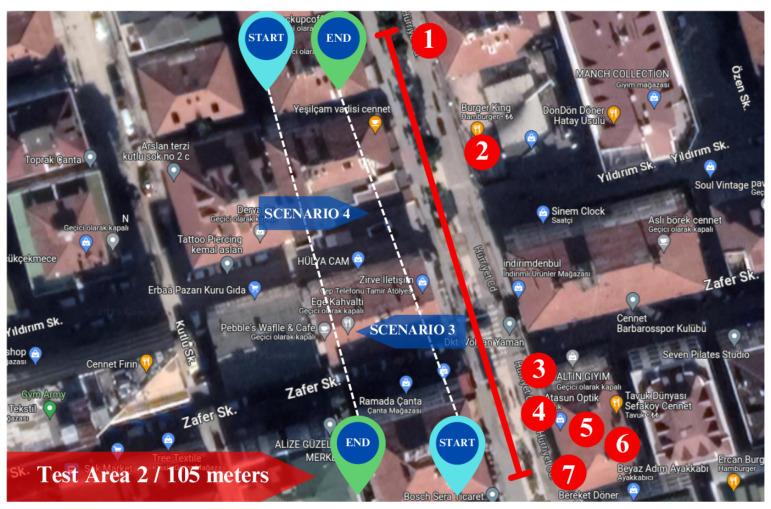
Start and end points of the robot for Test Area 2.

The second experiment was carried out in the street with pedestrians, namely Test Area 2. Figure 12 presents the testing with the start and end points. The test area is approximately 105 m long and obstructed by six- or seven-story buildings. Most of the street consists of tiles, while a small part is asphalt. The numerical labels in Figure 12 correspond to businesses referred to in Table 6. As seen in Figure 12, there are seven businesses, and each business has unique signboards with various fonts and backgrounds.

**Scenario 3**

The scenario took place in the evening, under ideal weather conditions. The experiment began at 09:34 PM and concluded at 09:38 PM. The starting point of the robot is at the middle of the business building marked with number seven, and its ending point is at the building denoted by number one in Figure 12. In this scenario, we obtain coordinates from the GPS module and the proposed system.

**Figure 13:**
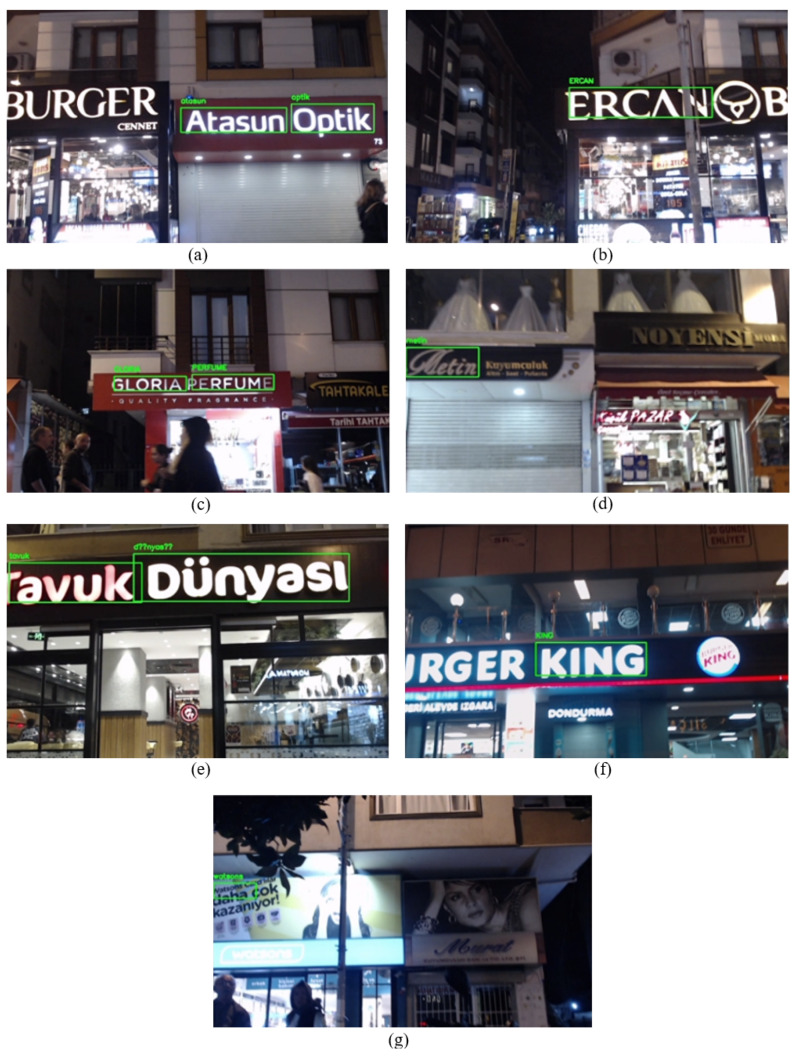
Detected and recognized business names by the proposed system in Scenario 3. (**a**) “Atasun Optik”, (**b**) “ERCAN BURGER”, (**c**) “GLORIA PARFUME”, (**d**) “Metin”, (**e**) “Tavuk Dünyası”, (**f**) “BURGER KING”, (**g**) “watsons”.

Each detected and recognized business name is depicted in Figure 13, in the sequence of their real location in the street. When the real-time video stream was examined, it was observed that the enhanced system performs well even in the evening under limited lighting conditions.

The enhanced system recognized the business names “Atasun Optik”, “ERCAN”, “GLORIA PERFUME”, “TAVUK” and “KING” many times from different angles. Moreover, the system recognizes the business names “Metin” and “Watsons” a few times. Due to the handwriting name of the business and constant objects in the street, the system encountered challenges in detecting and recognizing the label “Metin”. One important challenge in this scenario is lighting conditions, especially in night-time settings. Due to excessive light on the signboard in Figure 13 under option (g), the system could not detect the label “Watsons”. However, it detected and recognized the business’s name from the advertising section above the signboard, as seen in Figure 13 under option (g).

The recognized and corrected business names during Scenario 3 are presented in Table 7. The Easy OCR algorithm misrecognized one character for the business names “ERCAN”, “Metin”, and “watsons” and recognized them as “ERCA”, “Jetin” and “atsons”, respectively. Meanwhile, the rest of the business names are recognized without any difficulties. Due to the handwriting of the name “Metin” and the camera angle, the system failed to detect the character “M” properly, as can be seen in Figure 13 under option (d). In addition, due to the light in the signboards, EasyOCR failed to capture the characters “N” in “ERCAN” and “w” in “Watsons”. Finally, each misrecognized name was corrected successfully with Sequence Matcher.

For scenario 3, we consider the robot’s actual position as generated coordinates due to the lack of GPS signal sensitivity. Therefore, we measure distance difference in terms of GPS module error. As shown in Table 8, the distance difference between the GPS coordinates and the actual position of the robot when the robot was near the businesses “Atasun Optik”, “ERCAN BURGER”, “Metin” was around 43.25, 31.72, and 27.63 m, respectively. Further, the GPS signal improved its sensitivity when the robot approached the businesses “GLORIA PERFUME” and “Tavuk Dünyası”, resulting in a reduced distance difference of approximately 13 m. In the last business, we faced a lack of GPS signals. Therefore, the distance increases to 217 m. This scenario shows the importance of developing a localization support system that is not dependent on GPS signals.

**Figure 14:**
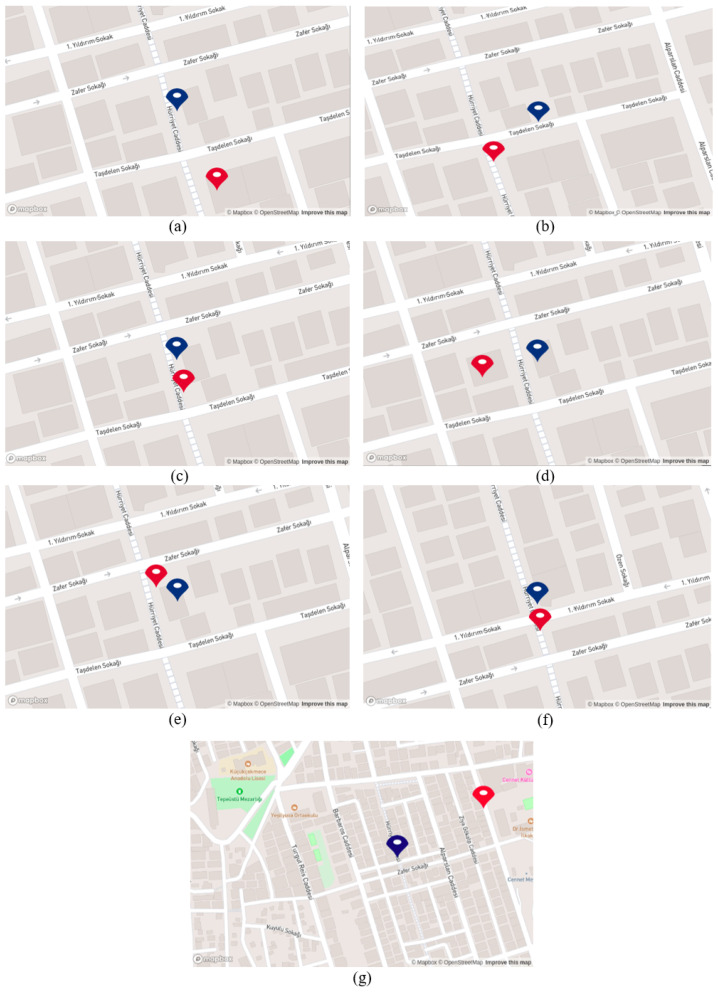
Display of GPS coordinates and generated coordinates on Mapbox during Scenario 3. (**a**) “Atasun Optik”, (**b**) “ERCAN BURGER”, (**c**) “GLORIA PARFUME”, (**d**) “Metin”, (**e**) “Tavuk Dünyası”, (**f**) “BURGER KING”, (**g**) “watsons”.

As mentioned before, we obtain coordinates from GPS and the proposed system during this scenario. Note that GPS coordinates are shown as red points, while the proposed system’s coordinates are shown as blue points. However, due to environmental factors such as tall buildings, we lost the GPS signal in some regions while the robot was in motion. In addition, when tracking the robot’s movement on the developed map, it was observed that the sensitivity of the GPS signal decreased. The red point updated itself over the buildings, whereas it should have been in the middle of the road, considering the robot’s actual position. As depicted in Figure 14, specifically under options (a), (c), (d), and (e), the red point is situated above the buildings, while the actual position of the robot is in the middle of the street “Hürriyet”. Additionally, in Figure 14 under option (g), the red point appears several streets above the robot’s actual position, showing the lack of robustness and low accuracy of GPS signals in this scenario. On the other hand, while the robot is moving in the street “Hürriyet”, option (a) to (g), it can be seen that the blue point is updating itself according to business places near the street, except for option (b). Here, the coordinate was expected to be near the street and in front of the building; however, the system generated the coordinate on the back side of the building. In general, updating its position near the street and in accordance with the business locations demonstrates the effectiveness of the proposed system in scenarios where GPS signals are either not sensitive or completely lost.

**Scenario 4**

This scenario was conducted during midday, under clear sunny weather conditions. The experiment started at 12:53 AM and ended at 12:58 AM. As in the second scenario, we could not obtain a signal from the GPS module due to environmental factors. Therefore, in this scenario, as the mobile robot is in motion, it updates its location on the map solely with the coordinates obtained from the proposed system, which is denoted with a blue point. We manually define a coordinate for the red point near the last business of the scenario to measure the difference with the blue point, in other words, with the generated coordinate. The starting and end points of the robot are opposite to Scenario 3.

**Figure 15:**
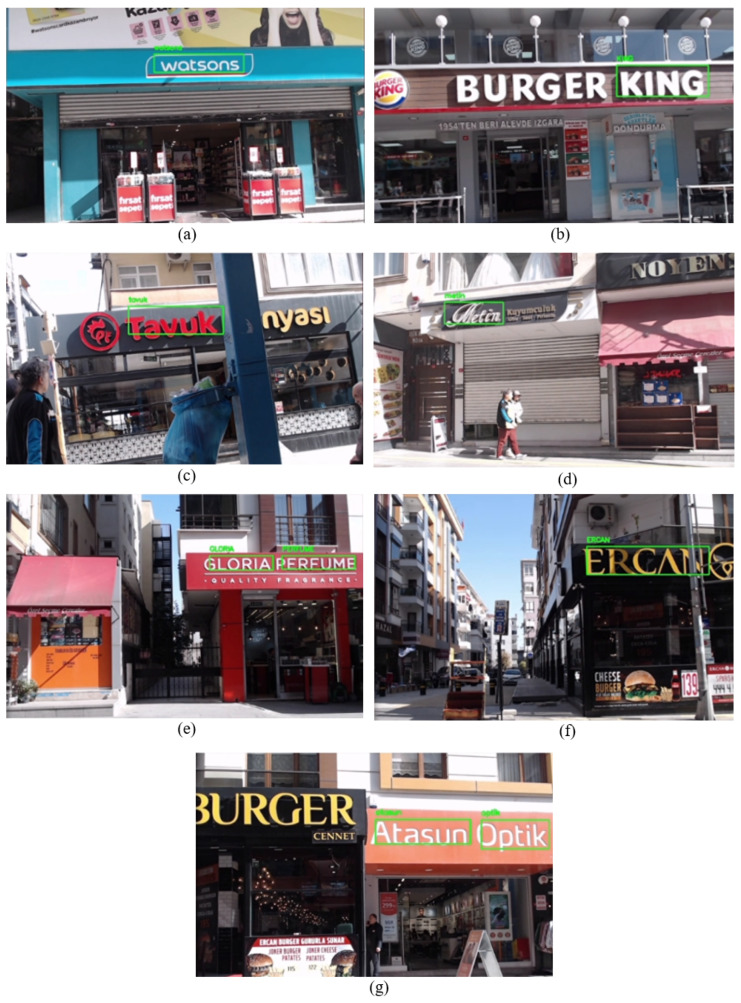
Detected and recognized business names by the proposed system during Scenario 4. (**a**) “watsons”, (**b**) “BURGER KING”, (**c**) “Tavuk Dünyası”, (**d**) “Metin”, (**e**) “GLORIA PERFUME”, (**f**) “ERCAN BURGER”, (**g**) “Atasun Optik”.

In contrast to Scenario 3, here, the enhanced system easily detected and recognized the business names “watson” as well as “Metin” dozens of times, as shown in Figure 15 under options (a) and (d). When the robot was in front of the business “Tavuk Dünyası”, some vibrations occurred due to the cobblestone surface of that region. Thus, the camera angle was altered, but the gimble quickly readjusted it to its standard position. Even in the challenging angles, the system recognizes the name “Tavuk Dünyası”, as can be seen under option (c). The system recognizes the rest of the businesses without any remarkable challenges. Some frames during the recognition of the names “watson”, “Burger King”, “GLORIA PARFUME”, “ERCAN BURGER” and “Atasun Optik” are demonstrated in Figure 15.

In Table 9, each recognized and corrected business name is shown with their similarity rate. The Easy OCR algorithm recognizes each business name except “Tavuk Dünyası” and “Metin”. The reason behind this was the vibration that occurred during the robot’s movement near the business “Tavuk Dünyası”. In addition, due to the limitations of the Easy OCR algorithm for handwritten characters, it could not recognize the character “M” when it comes to the business “Metin”.

When Table 10 was evaluated, it is clear that the distance difference with the red point decreases as the mobile robot approaches the endpoint. However, when the robot approaches the last two businesses that are placed in two adjacent buildings, the distance error does not decrease. This is due to the inaccurate coordinate placement on Google Maps, highlighting the limitation of the proposed system in scenarios where the reference coordinates are imprecisely defined on the map.

**Figure 16:**
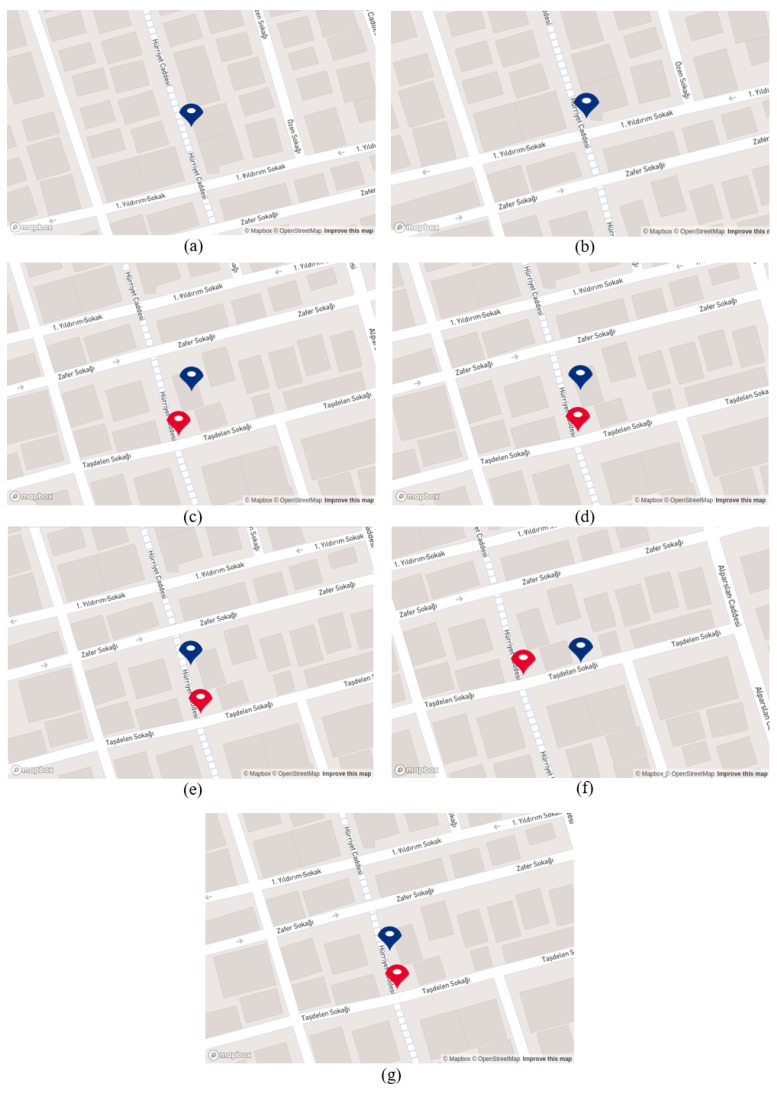
Display of GPS coordinates and generated coordinates on Mapbox during Scenario 4. (**a**) “watsons”, (**b**) “BURGER KING”, (**c**) “Tavuk Dünyası”, (**d**) “Metin”, (**e**) “GLORIA PERFUME”, (**f**) “ERCAN BURGER”, (**g**) “Atasun Optik”.

As mentioned before, the red point in this scenario was set to a constant position near the last business, “Atasun Optik”, to measure the generated system’s effectiveness when the mobile robot reaches the endpoint. Examining Figure 16 demonstrates that as the robot moves and recognizes business names, the blue point updates its position in accordance with the business places. However, when it recognizes the name “Ercan Burger” the generated coordinate is not located near the road or in front of the building; instead, it is situated on the back side of the building, as can be seen under option (f). This discrepancy is due to an inaccurate coordinate placement on Google Maps. The same discrepancy arises with the name “Atasun Optik”; the generated coordinate by the proposed system is not positioned in front of the business “Atasun Optik” but rather in front of a neighboring building, as seen in option (g).

#### 4.1.2. Case Study 3: Localization in Commercial District

**Figure 17:**
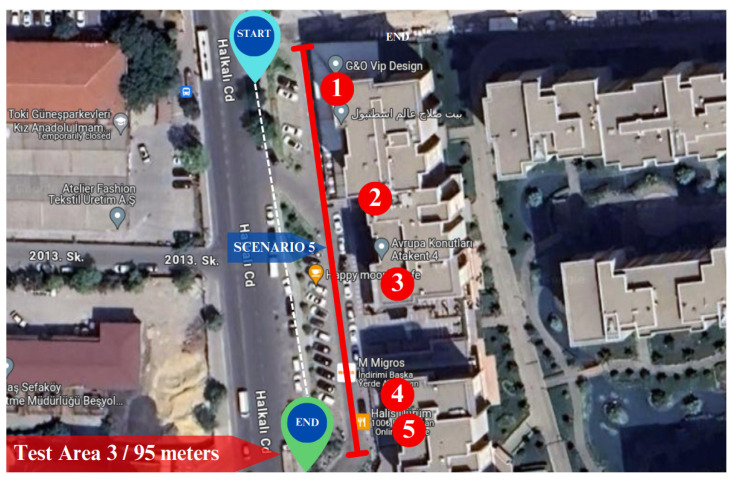
Start and end points of the robot for Test Area 3.

The final experiment was conducted in a commercial district in test Area 3. As shown in Figure 17, we designated starting and ending points for the motion of the mobile robot. The test Area 3 has a length of about 90 m. One side of test Area 3 is bordered by seven- or eight-story buildings, while on the opposite side, there is a road, followed by another set of multi-story buildings. The numbers in Figure 17 represent the businesses that are referred to in Table 11. Similar to the preceding testing areas, each business in this location features complex signboards presented in various fonts and set against diverse backgrounds.

**Scenario 5**

The experiment was conducted in the evening, under challenging conditions such as low light and a strong storm. To evaluate the performance of the proposed system in challenging conditions, including low light, a strong storm, and cobblestone terrain, the experiment was deliberately conducted during the evening hours on a surface with cobblestones. The robot’s initial position is at the start of the business building identified as number one, while its final destination is the building marked with the number five in Figure 17. In this experiment, we acquire coordinates from the GPS module and the proposed system. The experiments started at 05:33 PM and ended at 05:38 PM.

**Figure 18:**
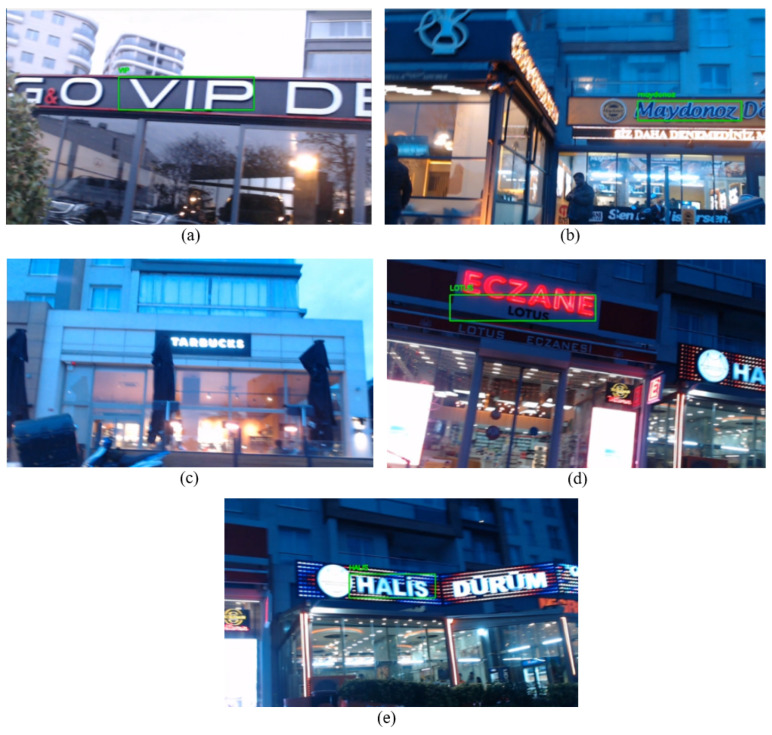
Detected and recognized business names by the proposed system during Scenario 5. (**a**) “G&O VIP”, (**b**) “Maydonoz”, (**c**) “Starbucks”, (**d**) “Lotus Eczane”, (**e**) “Halis Dürüm”.

In this experiment, the performance of the proposed system for text detection and recognition was robust despite challenges such as low light conditions, surface-induced vibrations leading to video stream blurriness, and the presence of a strong storm. The system effectively recognized all businesses within the test area except one name. Each recognized business name is demonstrated in Figure 18 under options (a), (b), (d), and (e). However, under option (c), the proposed system encountered difficulty in recognizing the business “STARBUCKS”. The reason behind this is the white light on the signboard name and blurriness caused by the storm, which altered the camera position. The reflection of the lights resulted in the characters appearing connected without clear spacing, posing a challenge for the system in recognizing the business name. On the other hand, the strong storm intermittently shifted the camera position throughout the experiments. Fortunately, the gimbal played a crucial role in restoring stability to the camera. The proposed system generally demonstrated remarkable performance in challenging conditions, including low light, a strong storm, and a cobblestone surface.

As indicated in Table 12, the EasyOCR algorithm accurately recognizes “VIP”, “Maydonoz” and “LOTUS” without missing any characters. However, it erroneously recognizes the name “HALIS” as “nHALIS”. Subsequently, this misrecognizing was corrected by the Sequence Matcher algorithm.

Table 13 compares the coordinates from the GPS module with the generated coordinates, emphasizing the differences in distance between the two sets of coordinates. Initially, when the robot was in front of business number 1, the distance difference was approximately 18 m. Subsequently, as GPS signals were lost, the distance difference increased to thousands of kilometers. At one point during the test when GPS signals briefly returned, the distance difference at that position was around 15 m, although this occurrence was short-lived.

**Figure 19:**
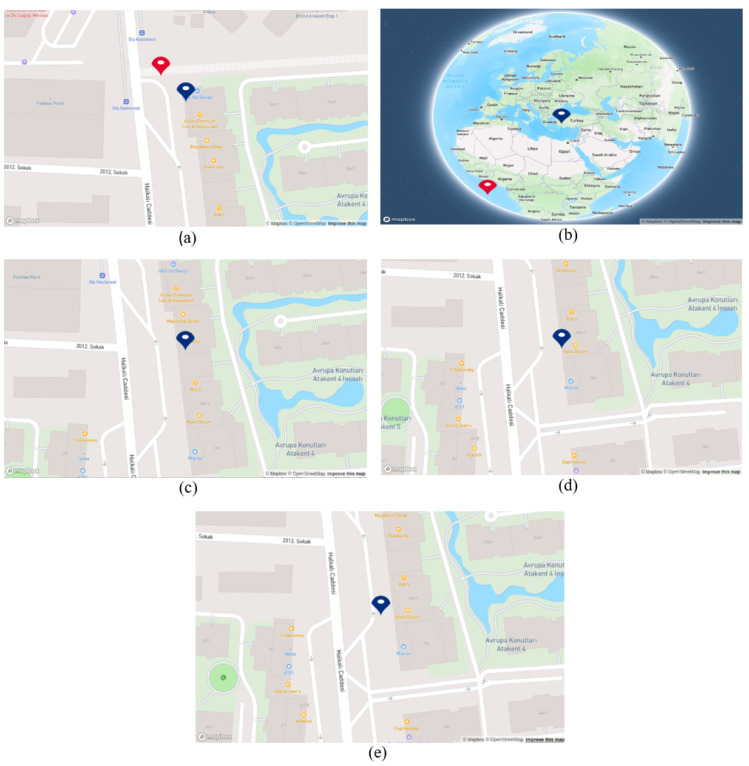
Display of GPS coordinates and generated coordinates on Mapbox during Scenario 5. (**a**) “G&O VIP”, (**b**) “Maydonoz”, (**c**) “Starbucks”, (**d**) “Lotus Eczane”, (**e**) “Halis Dürüm”.

As stated at the beginning of this scenario, during this experiment, while the mobile robot moving in the defined test area, we are acquiring coordinates from both the GPS and the proposed system. As can be seen in Figure 19 under option (a), when the mobile robot was in front of the business “G&O VIP”, the blue point accurately positioned itself in the front of the building, whereas the GPS coordinate, represented by the red point, was situated in the side street. Subsequently, when the mobile robot reached the business marked with number 2 in Table 11, the GPS signals were lost. By default, the module provided default coordinates, such as (0.0000, 0.0000). Consequently, the red point positioned itself near the intersection of Ecuador (latitude 0 degrees), as depicted in option (b). After the initial loss of GPS, there was a brief period during which GPS signals were available, but then signals were lost once again, and we were unable to acquire data for the rest of the test. However, the robot’s localization persisted using the generated coordinates from the proposed system until the end of the test. As evident in options (c), (d), and (e), the blue point consistently updated its position as the business names were recognized. This underscores the significance of developing a localization system that does not solely rely on GPS signals in urban areas.

### 4.2. System Evaluation

The localization performance of the proposed system is presented in Table 14. The accuracy described in Table 14 of the proposed system corresponds to Scenario 1, where good GPS signals were available. As highlighted in the manuscript, in the remaining scenarios, either no GPS signals were received, or the signals were noisy. Therefore, the performance evaluation of the proposed system’s accuracy was possible only in Scenario 1, where reliable GPS data were present. In other scenarios, we demonstrated that even when GPS signals were unavailable or when noisy signals were present, the proposed system enabled the robot to successfully localize itself.

When similar studies are examined in Table 14, most of the studies use few sensors in order to localize mobile robots, and the distance error between the reference and generated coordinates are given as minimum, maximum and average. In [29], the three different sensors are utilized. Even so, the average error rate was around 28 m. While it is crucial to conduct localization tests under various scenarios and in real-time, ref. [61] carried out their experiments through simulation and [60] performed their experiments via the video recordings. Although they achieved error rates of 4.23 m and 7.5 m, their studies lack real-time localization in urban environments. By employing three different sensors, ref. [27] achieved an average error rate of 4.12 m. While this is a closely comparable error rate to our work, it is noteworthy that we attained a marginally higher error rate of 4.20 m using only one sensor. Considering both the average and minimum error rates in our system, it is lower than the other studies. Consequently, the high accuracy and sensitivity of the proposed system was demonstrated through real-time experiments conducted in various test areas and light conditions. Taking into account both the average and minimum error rates in our system, they are lower than those presented in Table 14, except for the study by [27], where they achieved slightly lower error rates by a few centimeters. In general, the proposed system demonstrated high accurate localization through real-time experiments conducted in various test areas and light conditions.

In Table 15, we evaluated the operational efficiency of the proposed system, emphasizing its processing speed and average Frames Per Second (FPS). The processing speed refers to the duration elapsed from text detection to the reflection of coordinates on the map. The proposed system exhibited an average processing speed of approximately 2.5 s. Given the significance of time in the context of mobile robot localization, this performance can be considered remarkable. On the other hand, the proposed system, running on a laptop with limited computational capacity, achieved an average of 8 FPS.

### 4.3. Challenges and Discussion

During real-world experiments, several challenges were encountered in text detection, recognition, and localization, highlighting the complexity of operating in dynamic urban environments. In Scenario 1, the EAST text detection algorithm failed to detect PTT due to the closely spaced characters on the signboard, which were interpreted as a single merged character. Another challenge was the absence of a traditional signboard for A101. Despite this, the system detected the business name by recognizing the text near the store entrance. EasyOCR also struggled to recognize some businesses due to stylized fonts and closely positioned capital letters. However, these recognition errors were effectively corrected by integrating EasyOCR with the Sequence Matcher algorithm.

In Scenario 2, the mobile robot had to move closer to the businesses due to heavy traffic. Despite the closer distance, the system still failed to detect PTT because of the closely spaced characters on its signboard. Additionally, the higher camera angle compared to Scenario 1 caused further text detection and recognition difficulties. As a result, EasyOCR produced worse results than in the previous scenario. However, these issues were resolved by integrating EasyOCR with the Sequence Matcher algorithm, and the correct store names were recognized. Moreover, despite multiple attempts, no reliable GPS signals were obtained during this scenario, demonstrating the limitations of relying solely on GPS for localization, again. These circumstances highlight the importance of using a robust visual-based localization system that does not depend entirely on GPS signals.

The experiments in Scenario 3 were conducted during the evening, which presents distinct challenges. Several business names were handwritten, complicating the detection and recognition process. Additionally, nighttime conditions introduced some lighting issues. In some instances, excessive lighting caused glare on the signboards, preventing the system from directly detecting one business name. However, the system detected and recognized the business name from the advertising panel above the signboard. Moreover, GPS signals were inconsistent throughout the route, resulting in occasional inaccuracies where the robot’s position was displayed far from its actual location on the map. Despite these unreliable GPS signals, the proposed system effectively localized the robot. However, at one point, an incorrect coordinate caused temporary mislocalization. Once the following business was detected and recognized, the system promptly corrected the robot’s position, restoring accurate localization.

In Scenario 4, the experiments were conducted on the same street as in Scenario 3 but in the opposite direction and during the daytime. Consequently, lighting-related issues encountered during the nighttime experiment in Scenario 3 were not present in this scenario. However, at one point, ground-induced vibrations caused significant changes in the camera angle. This issue was mitigated by the gimbal mounted on the robot, which stabilized the camera and prevented further complications in text detection and recognition. Despite multiple attempts, reliable GPS signals could not be obtained in this scenario, similar to Scenario 3. As a result, the robot’s localization relied solely on the proposed visual-based system. As observed in the previous scenario, the system generated an incorrect coordinate at one point, leading to temporary mislocalization. However, once the next business was detected and recognized, the system quickly corrected the robot’s position, ensuring accurate localization.

In the final scenario, the primary challenges were the cobblestone surface and strong winds, which negatively impacted the system’s performance. The uneven cobblestone surface caused significant vibrations, leading to temporary blurriness in the video stream, which affected text detection and recognition. Additionally, the lighting conditions on certain signboards varied significantly, and in one instance, excessive lighting prevented the system from accurately detecting a business name. Moreover, the strong winds intermittently shifted the camera position during the experiments. However, this issue was effectively mitigated by the gimbal, which played a crucial role in maintaining camera stability and minimizing the negative effects of the storm. Throughout the experiment, GPS signals were either wholly unavailable or highly inconsistent. Although GPS signals were briefly received at a few points, they were quickly lost, which shows that relying on GPS for localization is unreliable.

## 5. Conclusions

This paper introduces a visual localization system designed for use in urban environments with low or blocked GPS signals. The proposed system consists of three steps: (1) Text detection-recognition, (2) generating coordinates, and (3) map development. For the first step, a text detection and recognition system was proposed. The EAST text detection algorithm was applied to detect texts in urban environments. The Easy OCR algorithm was employed to recognize the detected text. In addition, the applied text detection and recognition algorithms were enhanced by applying post-processing and word similarity algorithms. In the second step, recognized labels were fed to Places API to obtain POI data, especially coordinates, for a particular area. This process collected POI data for specific areas, including the names of businesses, their coordinates in terms of longitude and latitude format, operational status, etc. Moreover, an algorithm was developed to search the obtained data for the relevant label and return the most suitable coordinates using the Haversine formula in scenarios where GPS signals are blocked. In the last step, a map was developed using the Mapbox tool. The coordinates obtained from the GPS module and those generated by the proposed system are represented on the map with red and blue points. The red points demonstrate the coordinates of GPS, while the blue point shows the coordinates generated by the proposed system. Moreover, to evaluate the error rate of the proposed system, the distance between the generated coordinate and the GPS module coordinate is calculated by utilizing the Haversine Formula. The experiments carried out for text detection and recognition show that enhancing the system by applying post-processing and word similarity improves the recognition accuracy of the OCR algorithm. The proposed system’s localization performance with a delivery robot was evaluated in three different areas. Effective text detection and recognition performance, obtaining precise POI data, and developing an algorithm that returns respective coordinates contributed to the effective localization performance of the proposed system, resulting in an approximate error of around 4 m. Compared with other studies that employ methods like extended Kalman filter and Bayesian estimation, they reported similar error rates but relied on multiple sensors or were tested in simulations, increasing complexity. In contrast, our system uses only one sensor and was tested in real-world applications, making it more straightforward and practical. In addition, other studies that used more sensors reported higher error rates, such as 16 or even 28 m, highlighting the advantage of our approach. These results emphasize the significance of the proposed system in providing accurate and reliable localization with minimal sensor requirements in real-world urban environments.

In future work, we aim to address the issue of blurriness in the video stream caused by surface vibrations by employing advanced image processing filters and computer vision techniques. Additionally, we plan to reduce the error rate in the localization tasks of the proposed system. It will involve determining the robot’s pose and measuring the distance between the camera and the detected business name. These enhancements will enable more accurate localization of the robot within its environment.

## Figures and Tables

**Table 1:** Businesses and their addresses in Test Area 1.

Number	Business Name	Address
1	alBaraka	Cennet, Barbaros Cd. No: 73/B, 34290 Küçükçekmece/İstanbul
2	A101	Cennet, Barbaros Cd. No: 73, 34290 Küçükçekmece/İstanbul
3	YapıKredi	Cennet, Barbaros Cd. No: 71, 34290 Küçükçekmece/İstanbul
4	Ptt	Cennet, Barbaros Cd. 69B, 34290 Küçükçekmece/İstanbul
5	HALKBANK	Cennet, Barbaros Cd. No: 69, 34290 Küçükçekmece/İstanbul
6	VakıfBank	Cennet, Barbaros Cd. No: 65B, 34290 Küçükçekmece/İstanbul
7	KuveytTürk	Cennet Barbaros Cd. No: 65/A, 34290 Küçükçekmece/İstanbul
8	Ziraat	Cennet, Barbaros Cd. No: 63A, 34290 Küçükçekmece/İstanbul

**Table 2:** Recognized and corrected labels with the similarity rate in Scenario 1.

Business Name	Recognized Label	Corrected Label with Sequence Matcher	Word Similarity Rate (%)
alBaraka	alBaraka	alBaraka	100
A-101	A-101	A-101	100
YapıKredi	YapıKredi	YapıKredi	100
PTT	-	-	-
HALKBANK	HAIKBANK	HALKBANK	87.5
VakıfBank	Vakıf	Vakıf	100
KUVEYTTÜRK	KuUEYT	KUVEYT	83.3
Ziraat Bankası	7iraat	Ziraat	83.3

**Table 3:** Comparison of the GPS and generated coordinate in Scenario 1.

Business Name (Label)	GPS Coordinate	Generated Coordinate	Verification with Google Map	Distance Error (m)
alBaraka	lat:40.991123 long:28.778287	lat:40.9910718 long:28.778255	verified	6.05
A101	lat:40.991047 long:28.778276	lat:40.9910718 long:28.7782551	verified	3.27
YapıKredi	lat:40.990872 long:28.778350	lat:40.9908774 long:28.7783372	verified	1.23
PTT	lat:40.990768 long:28.778468	-	-	-
HALKBANK	lat:40.990647 long:28.778462	lat:40.9906612 long:28.7784283	verified	3.24
VakıfBank	lat:40.990503 long:28.778564	lat:40.9904952 long:28.7784983	verified	5.67
KUVEYTÜRK	lat:40.990434 long:28.778574	lat:40.9904682 long:28.7785097	verified	6.49
Ziraat Bankası	lat:40.99005 long:28.778730	lat:40.990093 long:28.778666	verified	5.43

**Table 4:** Recognized and corrected labels with the similarity rate in Scenario 2.

Business Name	Recognized Label	Corrected Label with Sequence Matcher	Word Similarity Rate (%)
Ziraat Bankası	iraa	Ziraat	80
KUVEYTTÜRK	KUUEYT	KUVEYT	83.3
VakıfBank	akıfB	Vakıf	80
HALKBANK	IHALKBA	HALKBANK	80
PTT	-	-	-
YapıKredi	Tapıkredi	YapıKredi	88.8
A-101	A-101	A-101	100
alBaraka	alBaraka	alBaraka	100

**Table 5:** Comparison of the GPS and generated coordinates in Scenario 2.

Business Name (Label)	GPS Coordinate	Generated Coordinate	Verification with Google Map	Distance Difference (m)
Ziraat Bankası	lat:40.991024803056135 long:28.7783132543341	lat:40.9902392 long:28.7786062	verified	90.75
KUVEYTTÜRK		lat:40.9904682 long:28.7785097	verified	64.05
VakıfBank		lat:40.9904952 long:28.7784983	verified	60.90
HALKBANK		lat:40.9906612 long:28.7784283	verified	41.57
PTT		-	-	-
YapıKredi		lat:40.9908774 long:28.7783372	verified	16.51
A101		lat:40.9910718 long:28.7782551	verified	7.15
alBaraka		lat:40.9910718 long:28.7782551	verified	7.15

**Table 6:** Businesses and corresponding addresses in Test Area 2.

Number	Business Name	Address
1	Watsons	Cennet, Hürriyet St. No: 62/B, 34290 Küçükçekmece/İstanbul
2	Burger King	Cennet, Hürriyet St. No: 60, 34290 Küçükçekmece/İstanbul
3	Tavuk Dünyası	Cennet, Hürriyet St. No: 56C, 34290 Küçükçekmece/İstanbul
4	Metin Kuyumculuk	Cennet, Hürriyet St. No: 56, 34290 Küçükçekmece/İstanbul
5	GLORIA PERFUME	Cennet, Hürriyet St. No: 54/A, 34290 Küçükçekmece/İstanbul
6	ERCAN BURGER	Cennet, Hürriyet St. No: 52/C, 34290 Küçükçekmece/İstanbul
7	Atasun Optik	Cennet, Hürriyet St. No: 52/B, 34290 Küçükçekmece/İstanbul

**Table 7:** Recognized and corrected labels with the similarity rate in Scenario 3.

Business Name	Recognized Label	Corrected Label with Sequence Matcher	Word Similarity Rate (%)
Atasun Optik	Atasun	Atasun	100
ERCAN BURGER	ERCA	ERCAN	88.8
GLORIA PERFUME	GLORIA PERFUME	GLORIA PERFUME	100
Metin	Jetin	Metin	80
Tavuk Dünyası	Tavuk	Tavuk	100
BURGER KING	KING	KING	100
watsons	atsons	watsons	88.6

**Table 8:** Comparison of the GPS and generated coordinates in Scenario 3.

Business Name (Label)	GPS Coordinate	Generated Coordinate	Verification with Google Map	Distance Difference (m)
Atasun Optik	lat:40.989670 long:28.780326	lat:40.9900175 long:28.7800944	Verified	43.25
ERCAN BURGER	lat:40.989714 long:28.780182	lat:40.98992640000001 long:28.7804342	Verified	31.72
GLORIA PERFUME	lat:40.989914 long:28.780121	lat:40.9900573 long:28.780811	Verified	16.28
Metin	lat:40.989959 long:28.779832	lat:40.9900289 long:28.7801479	Verified	27.63
Tavuk Dünyası	lat:40.990104 long:28.780083	lat:40.9900417 long:28.7802054	Verified	12.32
Burger King	lat:40.990328 long:28.779948	lat:40.9904457 long:28.7799321	Verified	13.16
Watsons	lat:40.991573 long:28.782114	lat:40.99064 long:28.77983	Verified	217.8

**Table 9:** Recognized and corrected labels with the similarity rate in Scenario 4.

Business Name	Recognized Label	Corrected Label with Sequence Matcher	Word Similarity Rate (%)
Watsons	watsons	watsons	100
BURGER KING	KING	KING	100
Tavuk Dünyası	Tavul	Tavuk	80
Metin	Ketin	Metin	80
GLORIA PERFUME	GLORIA PERFUME	GLORIA PERFUME	100
Ercan Burger	ERCAN	ERCAN	100
Atasun Optik	Atasun	Atasun	100

**Table 10:** Comparison of the GPS and generated coordinates in Scenario 4.

Business Name (Label)	GPS Coordinate	Generated Coordinate	Verification with Google Map	Distance Difference (meter)
Watsons	lat:40.98985981445177 long:28.78013560421319	lat:40.9906355 long:28.779864	verified	89.21
Burger King		lat:48.9964457 long:28.7799321	verified	67.35
Tavuk Dünyası		lat:40.9906417 long:28.7802054	verified	21.06
Metin Kuyumculuk		lat:40.9900289 long:28.7801479	verified	18.83
GLORIA PERFUME		lat:40.9900573 long:28.7800811	verified	14.43
ERCAN BURGER		lat:40.98992640000001 long:28.7804342	verified	26.13
Atasun Optik		lat:40.9900175 long:28.7800944	verified	17.87

**Table 11:** Businesses and corresponding addresses in Test Area 3.

Number	Business Name	Address
1	G&O VIP	Halkalı Caddesi, Atakent 4 No: 206L 34290 Küçükçekmece/İstanbul
2	Maydonoz	Halkalı Caddesi, Atakent 4 No: 206L 34290 Küçükçekmece/İstanbul
3	Starbucks	Halkalı Caddesi, No: 206 34290 Küçükçekmece/İstanbul
4	Lotus Eczane	Halkalı Caddesi, No: 206, D:E 34290 Küçükçekmece/İstanbu
5	Halis Dürüm	Avrupa Konutları 4, İnönü Mah. No: 206 34290 Küçükçekmece/İstanbul

**Table 12:** Recognized and corrected labels with the similarity rate in Scenario 5.

Business Name	Recognized Label	Corrected Label with Sequence Matcher	Word Similarity Rate (%)
G&O VIP	VIP	VIP	100
Maydonoz	Maydonoz	Maydonoz	100
STARBUCKS	-	-	-
LOTUS	LOTUS	LOTUS	100
HALIS	nHALIS	HALIS	87.5

**Table 13:** Comparison of the GPS and generated coordinates in Scenario 5.

Business Name (Label)	GPS Coordinate	Generated Coordinate	Verification with Google Map	Distance Difference (kms)
G&O VIP	lat:41.021457 long:28.791342	lat:41.021316 long:28.791462	verified	0.018
Maydonoz	lat:0.0000 long:0.0000	lat:41.02093 long:28.791677	verified	5405.025
STARBUCKS	lat:0.0000 long:0.0000	-	-	-
Lotus Eczane	lat:0.0000 long:0.0000	lat:41.0205738 long:28.7916786	verified	5404.995
Halis Dürüm	lat:0.0000 long:0.0000	lat:41.020566 long:28.791589	verified	5404.989

**Table 14:** Comparison of the proposed model and existing studies.

Research	Sensors	Method	Environment	Experiment Type	Error Rate (meters)
[29]	GPS/Compass/ Camera	Faster R-CNN	Outdoor	Real-Time	Average:28 Minimum:1
[27]	GPS, IMU, Visual Odometry	Extended Kalman Filter	Outdoor	Real-Time	Average:4
[60]	GPS/ Contour Action Camera	Image Retrieval Algorthim	Outdoor	Video Record	Average:7
[61]	Camera	Bayesian non-parametric estimation & particle filter	Outdoor	Simulation	Average:4
[62]	INS/GPS	Kalman Filter	Outdoor	Real-Time	Average:16
**Proposed**	**GPS/Camera**	**EAST+EASYOCR+SM** **&** **WEB MINING**	**Outdoor**	**Real-Time**	**Average:4** **Minimum:1**

**Table 15:** Performance evaluation of the proposed system in terms of FPS and processing time.

Scenario	Business	Processing Time (s)	Average FPS	Processing Time Average (s)
1	alBaraka	2.9	9.1	2.93
	A-101	3.1		
	YapıKredi	3.2		
	PTT	n/a		
	HALKBANK	2.8		
	VakıfBank	3.1		
	KUVEYTTÜRK	3.2		
	Ziraat Bankası	2.3		
2	Ziraat Bankası	2.7	7.5	2.6
	KUVEYTTÜRK	2.6		
	VakıfBank	2.8		
	HALKBANK	n/a		
	PTT	2.4		
	YapıKredi	2.8		
	A-101	2.5		
	alBaraka	2.3		
3	Atasun Optik	2.5	5.9	2.4
	ERCAN BURGER	2.5		
	GLORIA PERFUME	2.4		
	Metin	2.4		
	Tavuk Dünyası	2.3		
	BURGER KING	2.3		
	Watsons	2.3		
4	Watsons	1.8	5.10	2.1
	BURGER KING	1.8		
	Tavuk Dünyası	1.9		
	Metin	2.2		
	GLORIA PERFUME	1.9		
	ERCAN BURGER	2.3		
	Atasun Optik	2.5		
5	G&O VIP	2.4	8.5	2.4
	Maydonoz	2.5		
	STARBUCKS	n/a		
	LOTUS	2.6		
	HALIS	2.2		

## Data Availability

Data are contained within the article.
